# SARS-CoV-2: Repurposed Drugs and Novel Therapeutic Approaches—Insights into Chemical Structure—Biological Activity and Toxicological Screening

**DOI:** 10.3390/jcm9072084

**Published:** 2020-07-02

**Authors:** Cristina Adriana Dehelean, Voichita Lazureanu, Dorina Coricovac, Marius Mioc, Roxana Oancea, Iasmina Marcovici, Iulia Pinzaru, Codruta Soica, Aristidis M. Tsatsakis, Octavian Cretu

**Affiliations:** 1 Faculty of Pharmacy, “Victor Babes” University of Medicine and Pharmacy, 2nd Eftimie Murgu Sq., 300041 Timisoara, Romania; cadehelean@umft.ro (C.A.D.); iasminamarcovici@yahoo.com (I.M.); iuliapinzaru@umft.ro (I.P.); codrutasoica@umft.ro (C.S.); 2 Faculty of Medicine, “Victor Babes” University of Medicine and Pharmacy, 2nd Eftimie Murgu Sq., 300041 Timisoara, Romania; lazureanu.voichita@umft.ro (V.L.); tavicretu@yahoo.com (O.C.); 3 “Dr. Victor Babes” Clinical Hospital for Infectious Diseases and Pneumophthisiology, 300310 Timisoara, Romania; 4 Faculty of Dental Medicine, “Victor Babes” University of Medicine and Pharmacy, 2nd Eftimie Murgu Sq., 300041 Timisoara, Romania; roancea@umft.ro; 5 Department of Forensic Sciences and Toxicology, Faculty of Medicine, University of Crete, Heraklion, 71003 Crete, Greece; tsatsaka@uoc.gr

**Keywords:** SARS-CoV-2, remdesivir, chloroquine, hydroxychloroquine, lopinavir/ritonavir, convalescent plasma

## Abstract

SARS-CoV-2 (severe acute respiratory syndrome coronavirus 2) pandemic represents the primary public health concern nowadays, and great efforts are made worldwide for efficient management of this crisis. Considerable scientific progress was recorded regarding SARS-CoV-2 infection in terms of genomic structure, diagnostic tools, viral transmission, mechanism of viral infection, symptomatology, clinical impact, and complications, but these data evolve constantly. Up to date, neither an effective vaccine nor SARS-CoV-2 specific antiviral agents have been approved, but significant advances were enlisted in this direction by investigating repurposed approved drugs (ongoing clinical trials) or developing innovative antiviral drugs (preclinical and clinical studies). This review presents a thorough analysis of repurposed drug admitted for compassionate use from a chemical structure—biological activity perspective highlighting the ADME (absorption, distribution, metabolism, and excretion) properties and the toxicophore groups linked to potential adverse effects. A detailed pharmacological description of the novel potential anti-COVID-19 therapeutics was also included. In addition, a comprehensible overview of SARS-CoV-2 infection in terms of general description and structure, mechanism of viral infection, and clinical impact was portrayed.

## 1. Introduction

Since the beginning of the new decade of the 21st century—2020, humanity has been burdened by the emergence of a novel coronavirus known as SARS-CoV-2 (severe acute respiratory syndrome coronavirus 2), which caused a deadly outbreak of coronavirus disease (COVID-19) [1]. The first mention of this novel virus dates from the end of December 2019, and it is linked to a cluster of atypical pneumonia cases (27 cases) recorded in Wuhan, Hubei province, China [2,3]. The infection was declared a pandemic on 11 March 2020 by the World Health Organization (WHO) [4] and became an imperious public health concern.

**Figure 1:**
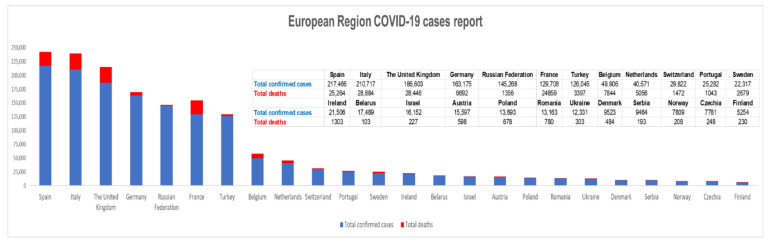
The European Region coronavirus disease (COVID-19) cases situation reported on 4 May 2020 according to World Health Organization (WHO) reports (in the graphic are presented only the countries with more than 5000 cases at that date) [8].

SARS-CoV-2 is the third highly pathogenic coronavirus that crossed the species barrier to cause fatal pneumonia in humans, after the SARS and MERS viruses causing the “severe acute respiratory syndrome” in 2002–2003 and the “Middle East respiratory syndrome” in 2012, respectively [1]. It has been reported that the SARS-CoV pandemic potentially led to up to 8000 cases of infection with an approximately 10% fatality rate in the early 2000s, while MERS-CoV produced over 1700 cases and an approximately 36% fatality rate later on [5]. Still, the recently discovered coronavirus elicits a greater rate of transmission, being already spread on all continents and encountering over 3,430,000 cases of infection up to the date of writing this article [6]. Therefore, to date of writing this article, the number of confirmed COVID-19 patients worldwide [7] was 3,435,894 with 239,604 reported deaths (Figure 1) [8] and, based on the trends observed in the last days, these numbers might be underestimated in the upcoming period.

SARS-CoV-2 pandemic represents the topic in focus in these latter days, and although notable progress was recorded in gaining knowledge about this fatal disease, there still are multiple gaps to fill for a full perspective. This review plans to offer a comprehensible overview of SARS-CoV-2 infection in terms of general description and structure, mechanism of viral infection, clinical impact, and investigational anti-COVID-19 therapy.

## 2. Severe Acute Respiratory Syndrome Coronavirus 2 (SARS–CoV-2)—A Brief Portrait

In the event of a viral infection, and particularly when the infection has pandemic potential, identifying the source of infection is decisive in controlling its spread [9]. To date, genome sequence analyses were performed and the first results confirmed the membership of SARS-CoV-2 to the family Coronaviridae, genus Betacoronavirus, and subgenus Sarbecovirus (which also includes SARS-CoV) [10], and granted the development of real time (RT)-PCR (polymerase chain reaction) diagnostic tests used for SARS-CoV-2 identification [11].

The members of the Coronaviridae family, Nidovirales order, are large enveloped viruses with a single-stranded positive-sense RNA [5,12]. These viruses exhibit phenotype and genotype differences and are grouped in four genera, as follows: alphacoronavirus (e.g., human coronavirus NL63 (HCoV-NL63), human coronavirus 229E (HCoV-229E), porcine transmissible gastroenteritis coronavirus (TGEV), porcine epidemic diarrhea virus (PEDV), and porcine respiratory coronavirus (PRCV)), betacoronavirus (e.g., SARS-CoV-2, SARS-CoV, MERS-CoV, bat coronavirus HKU4, mouse hepatitis coronavirus (MHV), bovine coronavirus (BCoV), human coronavirus OC43, and human coronavirus Hong University 1 (HCoV-HKU1)), gammacoronavirus (e.g., avian infectious bronchitis coronavirus (IBV)), and deltacoronavirus (e.g., porcine deltacoronavirus (PdCV) [5,12,13,14].

Coronaviruses (CoVs) are known to induce persistent and long-term severe health issues (respiratory, gastrointestinal tract infections, and central nervous system illnesses in humans and animals) owing to their ability to adapt to new environments through mutation and recombination, and to change host domain and tissue tropism [5,13]. A common feature of CoVs is their large genome consisting of 27 to 32 kb (the biggest genome of all RNA viruses) [5] as well as the variable number (6–11) of ORFs (open reading frames) that encode non-structural proteins (the first ORF accounts for 67% of the whole genome) as well as accessory and structural proteins (the remaining ORFs) [13].

**Figure 2:**
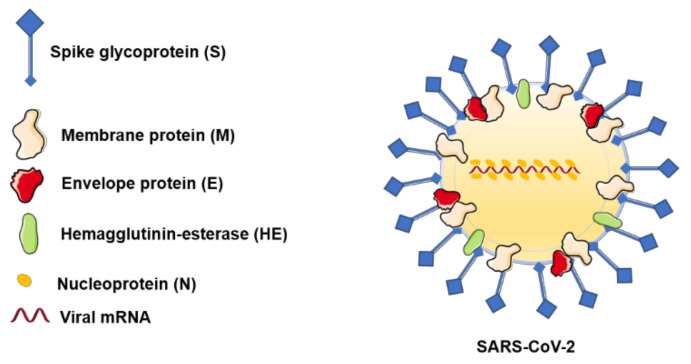
Severe acute respiratory syndrome coronavirus 2 (SARS-CoV-2) structure. This image contains Servier Medical Art elements, which are licensed under a Creative Commons Attribution 3.0 Unported License; https://smart.servier.com.

Four main viral structural proteins were described that were also detected in the SARS-CoV-2 structure (Figure 2): nucleocapsid protein (N), a helical capsid that contains the viral genome; matrix/membrane protein (M) and small envelope protein (E), both participatory in the virus assembly; and, finally, the spike surface glycoprotein (S), which intervenes in the virus entry within host cells [5,13]. The S, M, and E proteins are all embedded in the viral envelope, while the N protein is located in the core of the viral particle, forming the nucleocapsid [15]. The M proteins are among the most important and abundant proteins in the virion structure, being responsible for the virus shape. Their presence is critical as they play a key role, along with the E proteins, in orchestrating the assembly of the virus and in forming mature viral envelopes. The E proteins are found in small quantities within the viral particles, facilitating the release of the virions from the host cells. The N protein is required for RNA packaging into the viral particle during viral assembly [15]; some authors consider that the N proteins are also responsible for countering the host immune response and its defense mechanisms against infections [16].

Although its source is not yet confirmed, the newly emerged SARS-CoV-2 is considered to originate from the Chinese seafood markets [1]. Detailed comparative genomic sequence analyses were performed between SARS-CoV, SARS-like bat CoV, MERS-CoV, and SARS-CoV-2, reporting the following findings: SARS-CoV-2 presents a higher homology in terms of the whole genome sequence (encoded proteins of pp1ab, pp1a, envelope, matrix, accessory protein 7a, and nucleocapsid genes) with SARS-like bat CoV when compared with SARS-CoV. These data confirm the zoonotic origin of SARS-CoV-2 [10,13] and support the recent theory that the transmission chain started from bats to humans, although the intermediate hosts remain undetermined [17]. The transmission of the 2002–2003 SARS-CoV to humans was reported to occur from market civets, while that of the 2012 MERS-CoV emerged from dromedary camels; however, further investigations associated the pathogenic SARS and MERS viruses with bats [1]. Thus, these three viruses are thought to have a zoonotic origin (1), as wild animals are considered natural reservoir hosts playing a crucial role in transmitting various pathogens, including coronaviruses [9].

Nevertheless, both SARS-CoV and SARS-CoV-2 present similar receptor-binding domain structures [1] and a 79.5% homology [17]. Moreover, the main protease is highly preserved between SARS-CoV-2 and SARS-CoV with a 96% overall similarity [1]. In contrast, the resemblance rate between the new coronavirus and MERS-CoV was only 50% [15]. Even though the percentage of similarity between SARS-CoV-2 and SARS-CoV was quite high in the nucleotide sequence, the novel pathogenic coronavirus displays significant specific features, mainly in the accessory proteins located in the 3′-terminus of the genome: absence of the 8a protein, a longer 8b protein consisting of 121 amino acids when compared with 84 in SARS-CoV, and a shorter 3b protein of only 22 amino acids. The new SARS-CoV-2 also differs from the other coronaviruses by encoding an additional hemagglutinin (HE) glycoprotein that possesses acetyl-activity, which might enhance the cell entry and pathogenesis of the virus [15,16]. The first genome sequence of the new SARS-CoV-2 consisting of 29,903 bp is currently available and has been since January 2020 in the NIH GenBank database, as the reference sequence, under the accession number NC_045512 [18]. The attempt to study significant features/mutations related to genome sequences related to different geo-located SARS-CoV-2 strains is a continuous and imperative work. This ongoing effort is highlighted by the large number of GenBank repository entries (over 8000) available under the SARS-CoV-2 genome sequence as of May 2020. This achievement is important and has aided various studies that compare occurring variations in the genomic sequence to detect mutations [19], which can also impact the sensitivity of some of the widely used diagnostic methods such as RT-PCR [20]. These data shed some light regarding the genome of SARS-CoV-2, but the real impact of these differences in terms of pathogenesis is still under investigation [13].

### 2.1. Mechanism of SARS-CoV-2 Viral Infection

A major event in the viral infection is represented by the binding of viral particles to cellular receptors located on host cells’ surface, a function that is executed by spike S glycoprotein [21,22]. The S glycoprotein is a highly exposed protein [22], projecting from the virion surface and giving its royal crown aspect under electronic microscopy [15]. Thanks to their position, the S proteins are the main targets for design of therapeutics and vaccines [22]. In light of the major roles played by spike S glycoprotein in viral infection and the impact of CoVs on human health, previous studies focused on elucidating the structure of this glycoprotein as a potential target for antiviral therapies. S glycoprotein resembles a clove-shape trimer with three structural parts: (1) a large ectodomain organized in two subunits—S1 and S2—responsible for viral and host membrane fusion and transfer of the genome into the host cell; (2) a single-pass transmembrane anchor; and (3) a short tail located intracellularly [5,22]. The S1 subunit is involved in the recognition of the cellular receptor and presents two domains: (i) NTD (N-terminal domain) and (ii) CTD (C-terminal domain), which act as receptor-binding ligands ensuring viral attachment [5,21,22]. Besides the key role of spike surface glycoprotein in the viral penetration into the host cells, it is also responsible for inducing immune responses from the host and establishing viral host domain and tissue tropism [5]. SARS coronaviruses, including SARS-CoV-2, predominantly infect airway and alveolar epithelial cells, vascular endothelial cells, and macrophages [23], but the tissue affinity of CoVs generally depends on the ability of S protein to interact with the receptors expressed by the host cells [15].

Previous studies elucidated the CoVs’ pattern to recognize host cellular receptors: SARS-CoV identifies the zinc peptidase angiotensin-converting enzyme 2 (ACE2) via S1 C-terminal domain (also known as receptor binding domain—RBD), whereas MERS-CoV identifies serine peptidase and dipeptidyl peptidase 4 (DPP4), also using the S1 C-terminal domain [5,21]. Recent studies showed that SARS-CoV-2 exhibits a 10–20 times [17] stronger affinity for human ACE2 receptor when compared with SARS-CoV and the binding is also performed via the C-terminal domain [21,24]. Up until the submission of this manuscript, four different studies reported the 3D structure of the SARS-CoV-2 RBD–ACE2 complex. All structures were deposited in the RCSB (Research Collaboratory for Structural Bioinformatics) Protein Data Bank (PDB) under the following PDB IDs: 6VW1, 6M0J, 6LZG, and 6M17, and show useful insight in the binding interactions formed between the two proteins [21,25,26,27]. According to some researchers, angiotensin-converting enzyme 2 is not only the cellular entry locus for CoVs, but it also regulates both the cross-species and human-to-human transmissions of the virus [28]. ACE2 is physiologically expressed on the I and II type alveolar epithelial cells of the human lung, but also by other cells; therefore, SARS-CoV-2 binding to the receptor might cause cellular damage, a series of systemic reactions, and even death [17].

**Figure 3:**
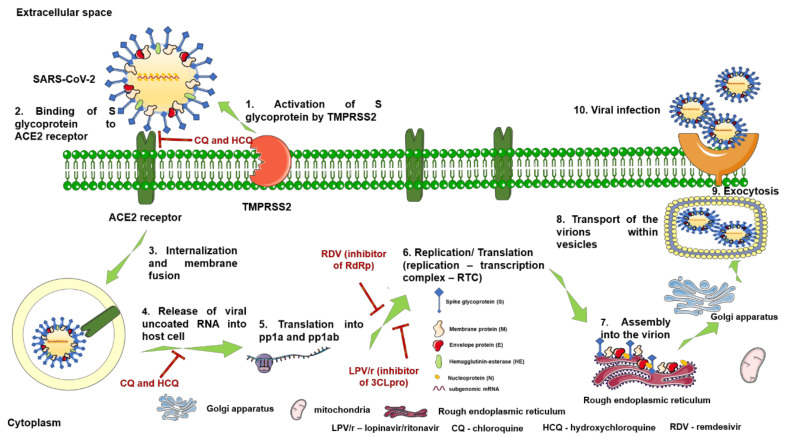
Mechanism of SARS-CoV-2 viral infection: (1) activation of S glycoprotein by transmembrane protease serine 2 (TMPRSS2); (2) activated S glycoprotein binds to the angiotensin-converting enzyme 2 (ACE2) receptor located on human cells surface (target for CQ—chloroquine and HCQ—hydroxychloroquine); (3) internalization and viral membrane fusion; (4) release of the uncoated RNA into the host cell (target for CQ and HCQ); (5) translation into the replicase polyproteins pp1a and pp1ab; (6) formation of replication–transcription complex (RTC) involved in replication and translation of structural proteins and synthesis of subgenomic mRNA, a process that occurs in the cytoplasm of the host cell; (7) assembly of the newly form viral RNA and the structural proteins to form the virion (in the rough endoplasmic reticulum and Golgi apparatus); (8) transport of the virions via vesicles that fuse with the plasmatic membrane; (9) release of the virus in the extracellular space via exocytosis; and (10) spread of the virus and viral infection. This image contains Servier Medical Art elements, which are licensed under a Creative Commons Attribution 3.0 Unported License; https://smart.servier.com.

The invasion of CoVs (Figure 3) into the human cells is a complex process that comprises the following steps: (i) spike S glycoprotein acquires a homotrimer form noticeable from the viral surface; (ii) cleavage of S glycoprotein at the boundary between S1 and S2 subunits by the cell surface-associated transmembrane protease serine 2 (TMPRSS2) [29]; (iii) stabilization of the prefusion state of the membrane-anchored S2 subunit that possesses the fusion entity by S1 subunit; (iv) activation of protein for membrane fusion with irreversible conformational modifications by the host proteases cleavage at the S2‘ site; and (v) virus–cell fusion and transfer of viral genome into the host cell [22]. Further, the uncoated RNA translates two polyproteins (pp1a and pp1ab) that encode non-structural proteins and form a replication–transcription complex (RTC). RTC replicates and synthesizes subgenomic RNAs that encode accessory proteins and structural proteins. Finally, the newly formed genomic RNA, nucleocapsid proteins, and envelope glycoproteins assemble to form virion-containing vesicles able to fuse with the plasma membrane and release the new virus [28,30].

### 2.2. Clinical Impact of SARS-CoV-2

On the basis of the existing literature, COVID-19 can be defined as an acute respiratory syndrome that affects primarily the lungs, causing pneumonia that can progress to a more severe stage; that is, acute respiratory distress syndrome (ARDS), multiorgan failure, and even death [31]. Lungs were considered the main target for SARS-CoV-2 infection, but other injured organs were also described after encounter with the virus, such as esophagus, small intestine, colon, stomach, kidney, and testis, on the account of the ACE2 receptors that are expressed at their level [31].

Initial evidence supported the hypothesis that the transmission of SARS-CoV-2 occurs via droplets, respiratory fluids, and direct contact, but recent data presented novel modes of virus spread such as fecal–oral route, via bodily fluids, and transmission through environmental surfaces [32,33].

Clinically, four stages of SARS-CoV-2-induced infection were described: the asymptomatic carrier state, mild-to-moderate (81% of cases), severe (14% of cases), and critically ill (5% of cases) form [9,34]. Another classification of infection stages was made according to the results obtained by non-contrast enhanced chest computed tomography (CT), as follows: early stage (0–4 days) with ground glass opacities (GGOs) in the lower lung lobes; progressive stage (5–8 days) with bilateral dissemination of infectious process and diffuse ground glass opacities; peak stage (9–13 days) with dense consolidation and residual parenchymal bands; and absorption stage (>14 days) with gradual resolution of the lesions observed, as a sign of recovery [32].

The asymptomatic patients infected with SARS-CoV-2 are difficult to identify as they do not manifest any symptoms; still, they are able to spread the virus [3] through droplets, respiratory secretions, and direct contact [28].

**Figure 4:**
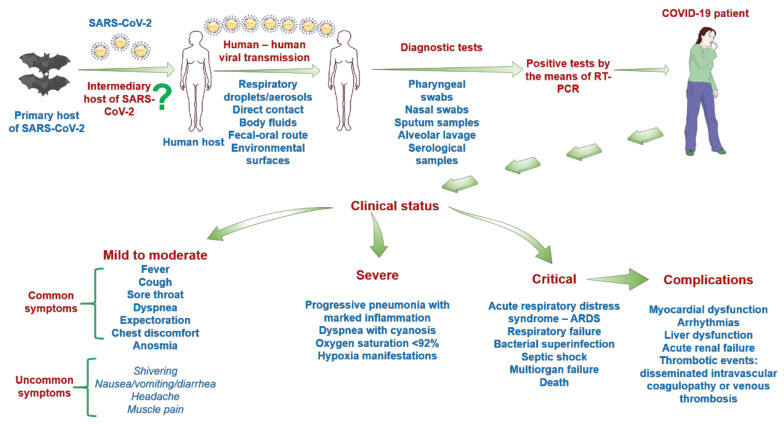
Schematic overview of the steps involved in SARS-CoV-2 infection from the first contact with the virus until the final phase—death or recovery. This image contains Servier Medical Art elements, which are licensed under a Creative Commons Attribution 3.0 Unported License; https://smart.servier.com.

The symptomatic patients with mild-to-moderate to severe and rapidly progressive disease present a complex symptomatic panel from mild to fatal manifestations (Figure 4), data that are constantly updated [9]. The symptoms usually appear within 2–14 days after viral exposure [35], but this onset interval and the symptomatology are highly patient-dependent. This myriad of symptoms could be explained by the broad expression of ACE2 receptors within the body: on alveolar type-1 and type-2 pneumocytes and lung stem/progenitor cells, on vascular endothelial cells [36,37], in terminal ileum intestinal epithelial cells (high expression), in the colon and in the stomach (a lower expression) [29], in the bile duct epithelial cells (a 20× higher expression than in hepatocytes) [38], in the kidneys and testis (about 100-fold increased expression than in the lungs) [31], in cardiac myocytes, and in cells of the vascular endothelium [36,39]. The mild-to-moderate form of the infection is characterized by the following symptoms: flu-like symptomatology (fever, dry cough, runny nose, and fatigue), dyspnea, expectoration, chest discomfort, respiratory distress, and lymphocytopenia, which are labeled as common symptoms, but some uncommon ones were also described, such as shivering, throat pain, anosmia, headache, joint pain, nausea, vomiting, and diarrhea [17,29]. All patients exhibiting mild-to-moderate forms present abnormalities in chest computed tomography (CT) images [17]. At the time of writing the present review, anosmia was considered an uncommon symptom of SARS-CoV-2 infection, but according to the latest studies, this symptom became a frequently reported symptom of the infection in association with dysgeusia [40,41].

In the case of the severely and critically ill COVID-19 patients, the symptomatology is quite diverse, consisting of the following: progressive pneumonia with marked inflammation, status that might deteriorate to respiratory failure, pulmonary edema, acute respiratory distress syndrome (ARDS), bacterial superinfection, septic shock, multiorgan failure, and patient death [29]. Other complications were also noticed in these patients: myocardial dysfunction, arrhythmias, and acute renal failure [24].

Apart from the data provided above, emerging evidence indicates digestive disorders in COVID-19 patients, such as anorexia (most frequent symptom in adults—Chinese studies), diarrhea (common both in adults and children), vomiting (more common in children), gastrointestinal bleeding, abdominal pain, and intestinal flora disorders [33], as well as liver injury with abnormal values of hepatic enzymes [42] and thrombotic events [39].

It is hypothesized that the digestive disorders, liver injury, acute respiratory distress syndrome (ARDS), and multiorgan failure related to COVID-19 have a common triggering factor—“the cytokine storm” [29,33,42].

The virus–cell interactions and the viral rapid replications trigger the release of multiple pro-inflammatory cytokines and chemokines [23]. The inflammatory markers observed in COVID-19 patients include interleukin-1 (IL-1), interleukin-2 (IL-2), interleukin-4 (IL-4), interleukin-6 (IL-6), interleukin-7 (IL-7), interleukin-10 (IL-10), interleukin-12 (IL-12), interleukin-13 (IL-13), interleukin-17 (IL-17), fibroblast growth factor (FGF), granulocyte-macrophage colony stimulating factor (GM-CSF), interferon gamma (IFN-γ), granulocyte-colony stimulating factor (G-CSF), interferon-γ-inducible protein (IP10), monocyte chemoattractant protein (MCP1), macrophage inflammatory protein 1 alpha (MIP1A), platelet derived growth factor (PDGF), tumor necrosis factor (TNF-α), and vascular endothelial growth factor (VEGF) [28,43]. Additionally, SARS-CoV-2 infection may cause cell pyroptosis within macrophages and lymphocytes, which also leads to the release of large amounts of pro-inflammatory factors [23]. Thus, CoVs induce a local aggressive inflammation that may cause massive epithelial and endothelial cell apoptosis and vascular leakage, leading to respiratory injury [23]. Interestingly, some studies suggest that the S viral protein–ACE2 complex is directly involved in the inflammatory responses induced by the SARS-CoVs. S protein can downregulate ACE2 receptors, leading to the loss of their pulmonary functions, which are, unfortunately, still unknown. ACE2 dysregulations might cause dysfunction of the renin-angiotensin system as well, enhancing inflammation and vascular permeability. Moreover, the ACE2 shedding has been associated with TNF-α production [23].

Even if the immune response is vital for the control and resolution of CoVs-induced infections, it can also lead to immunopathogenesis [28]. Therefore, the second pathological mechanism of the SARS viruses is related to the anti-S protein-neutralizing antibodies (anti-S-IgG) released by the host in order to fight infection [23]. The antibody-dependent enhancement related to viral infection is a paradoxical process characterized by the presence of virus specific antibodies that augment viral entry within host cells and replication of the pathogen, leading to an exacerbation of the disease [44]. In vivo studies confirmed that the presence of the anti-spike protein antibodies not only induced viral suppression, but also caused severe acute lung injury in the early stages of SARS-CoV infection. It is believed that the antibody-dependent injuries are owing to their ability to enhance inflammatory responses from macrophages. Therefore, considering the mechanisms described above, Fu Y and co-workers presented two inflammatory stages mediated by the SARS-CoVs: (i) the primary stage occurs shortly after the viral infection, but prior to the appearance of neutralizing antibodies when the inflammatory responses are the consequence of the interaction between the spike proteins and the ACE2 receptors; and (ii) the secondary stage, which begins with the appearance of the antibodies and the generation of the adaptive immunity that can diminish viral replication or trigger inflammatory responses and cause severe lung injury [23].

The plethora of symptoms common to COVID-19 patients are endorsed by several laboratory abnormalities, such as lymphopenia, augmented values for lactate dehydrogenase and inflammatory markers: C-reactive protein, D-dimer, ferritin, and IL-6 (interleukin-6—marker for severe pathology and procoagulant profile) [39].

An intriguing finding, which still remains unclear, is the decreased susceptibility of children to SARS-CoV-2 infection as compared with the adult population. Even though an age-dependent susceptibility to SARS-CoV-2 infection was established, a valid explanation for these disparities is still lacking [45]. However, several hypotheses were drawn in this regard, as follows: (i) the mild-to-moderate clinical pattern observed in children could be explained by the immunization schedule, which might confer cross-protection to SARS-CoV-2 owing to the presence of a high titer of antibodies in children’s blood; (ii) a lower rate of exposure to infected population; (iii) a small number of tests performed on children owing to their absent or mild symptomatology; and (iv) the expression of ACE2 enzyme is elevated in later childhood, which could be considered a mechanism of protection for the children [46,47].

## 3. Drugs Recommended in COVID-19 Therapeutic Guidelines 

Up to date, neither an effective vaccine nor SARS-CoV-2-specific antiviral agents have been approved, and the management of coronavirus disease (COVID-19) focuses mainly on supportive care and symptomatic treatment [17,35,48].

Taking into consideration the global threat posed by the SARS-CoV-2 pandemic, imperative collaborative solutions were proposed in order to find a treatment against this unpredictable virus, such as the “SOLIDARITY” clinical trial for COVID-19 treatment, a study of great ampleness launched by WHO on 20th of March [49] and the scientific collaboration of a considerable number of researchers from different countries for the development of a COVID-19 vaccine [50]. At present, there are over 1300 studies recorded as clinical trials for COVID-19 in the ClinicalTrials.gov database [51], information that can also be accessed via the WHO Trial Registry Network on the International Clinical Trial Registry Platform (ICTRP) [52].

Considering the urgent need to find a treatment for COVID-19, two approaches were nominated: (i) a short term approach with immediate results—administration of approved drugs (originally introduced for other pathologies) with a high safety profile that showed promising in vitro results against SARS-CoV, MERS-CoV, and SARS-CoV-2 combined with convalescent plasma treatment (agents tested in ongoing clinical trials); and (ii) a long term approach—development of novel antiviral agents and vaccines against SARS-CoV-2.

On the basis of the literature data and the Chinese observational notes, interim COVID-19 treatment guidelines were created, guidelines that were adapted to each country and are constantly updated (Table 1) [53,54,55].

The therapeutic drugs mentioned in the interim guidelines for COVID-19 treatment (Table 1) were also selected for the SOLIDARITY trial: remdesivir (RDV), an experimental antiviral; chloroquine (CQ), an antimalarial agent (or hydroxychloroquine—HCQ); a combination of lopinavir and ritonavir (LPV/r), an anti-HIV medication; and lopinavir + ritonavir + interferon-beta (an antiviral and modulator of the immune system that acts by suppressing pro-inflammatory cytokines) (see Table 2) [56].

### 3.1. Remdesivir—RDV (GS-5734)

**Figure 5:**
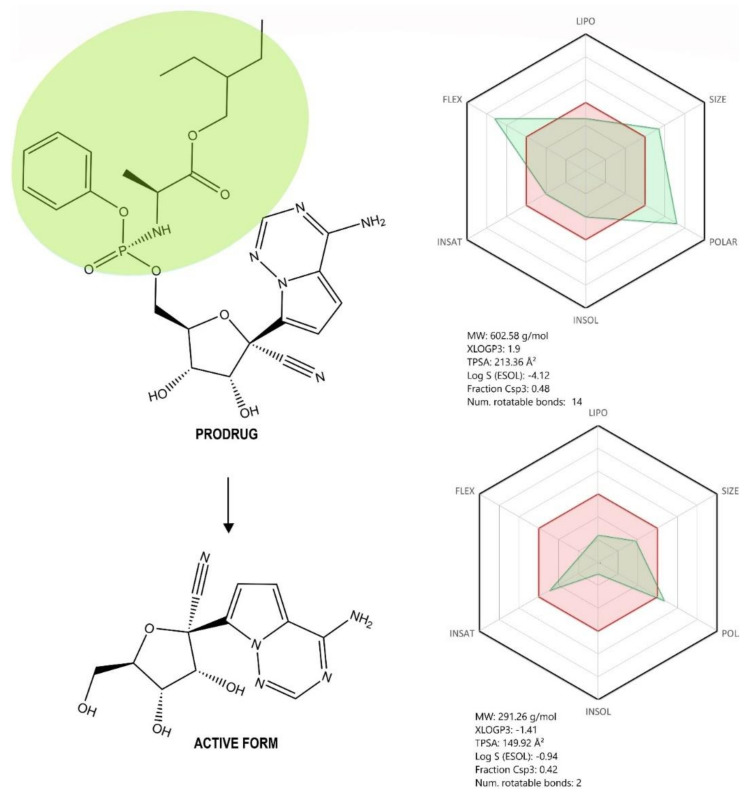
Chemical structure of the prodrug Remdesivir (RDV) and its active form GS-441524. Absorption, distribution, metabolism, and excretion (ADME) profile was achieved using the free web tool SwissADME; the red highlighted area represents the suitable physicochemical space for oral bioavailability covering value intervals for LIPO (lipophility): −0.7 < XLOGP3 < +5.0, SIZE: 150 g/mol < MV < 500 g/mol, POLAR (polarity): 20Å^2^ < TPSA < 130Å^2^, INSOLU (insolubility): 0 < Log S (ESOL) < 6, INSATU (insaturation): 0.25 < Fraction Csp3 < 1, FLEX (flexibility): 0 < Num. rotatable bonds < 9, whereas the overlapped green highlighted area shows the calculated ADME profile for the molecule [63].

Remdesivir (2-Ethylbutyl (2S)-2-{[(S)-{[(2R,3S,4R,5R)-5-(4aminopyrrolo[2,1-f][1,2,4]triazin-7-yl)-5-cyano-3,4-dihydroxytetrahydrofuran-2-yl] methoxy}(phenoxy) phosphoryl] amino} propanoate) (Figure 5, see brief description in Table 2) is considered at this point the most promising treatment for SARS-CoV-2 infection on the basis of the latest outcomes acquired within the phase III clinical trials sponsored by the manufacturer [57] and the National Institute of Allergy and Infectious Diseases (NIAID) in the Adaptive COVID-19 Treatment Trial (ACTT) [58].

RDV is the monophosphoramidate prodrug of C-adenosine nucleoside analogue GS-441524 (1′-cyano 4-aza-7,9-dideazaadenosine C-nucleoside—a compound that is prescribed as therapy against infectious peritonitis in cats and felines, a disease determined by a feline coronavirus) [59,60]. This drug was developed by Gilead Sciences, Inc. as an antiviral candidate against the Ebola virus, but it also proved to be highly efficient in vitro against multiple RNA viruses, both negative sense, paramyxoviridae (parainfluenza type 3 virus, measles and mumps viruses, and nipah virus, among others) and pneumoviridae (respiratory syncytial virus), and positive sense viruses, coronaviridae (SARS-CoV, MERS-CoV, mouse hepatitis virus (MHV), and HCoV-OC43, among others) [60,61,62].

Structurally, RDV is a nucleoside analogue, which closely resembles AMP (adenosine monophosphate); important structural differences occur both in the nucleobase region, where the purine ring is replaced by a pyrrolo[2,1-f][1,2,4]triazine, and the ribose region, where a cyanide group is present in the fifth position. Moreover, the phosphate group is attached to a phenoxi group and an α-Alanine-2-ethyl-butyl ester, thus increasing the molecule’s lipophilicity as well as its degree of structural flexibility (Figure 5). Given its molecular shape, RDV acts as ATP (adenosine triphosphate) competitive inhibitor, targeting an enzyme involved in the viral genome replication, namely RNA-dependent RNA polymerase (RdRp) [31,48,61].

RDV is actually a prodrug metabolized intracellularly in different human cells [31,60,61] following the subsequent action of various enzymes (esterases and phosphoramidases), and is converted to the monophosphate form of RDV’s active metabolite, GS-441525, which is further phosphorylated by nucleoside-phosphate kinases to its active triphosphate form, interfering with the action of the viral RdRp of Ebola virus via insertion into the newly nascent viral RNA chain and disruption of the virus transcription [61,64,65,66]. RDV exerts a similar mechanism of action in coronaviruses by triggering the inhibition of RdRp complex and the premature termination of the viral RNA chain [31,48]. Moreover, RDV was able to bypass the proofreading 3′-5′ exoribonuclease (ExoN), a unique feature of CoVs, responsible for the decreased activity of various nucleoside analogues used as antivirals [59]. Recent studies identified the architecture of RdRp complex in SARS-CoV-2, the target site for RDV, an important step for a better understanding of the antiviral mechanism of action of RDV in SARS-CoV-2 infection [67,68,69].

In order to investigate whether this mechanism of action is also responsible for the antiviral effect on SARS-CoV-2, a recent study, using computational methods, constructed a homology model of SARS-CoV-2 NSP12 RdRp (nonstructural protein 12 of the RdRp complex) [70]. On the basis of this model, the authors assessed ATP/RDV binding with SARS-CoV-2 NSP12 by means of molecular docking, using a previous co-crystal structure of the poliovirus RdRp. The study showed that the difference between the calculated binding energy of RDV triphosphate and that of ATP was significantly large, concluding that ATP can be blocked out of the binding site when RDV triphosphate is locally present, deeming that RDV could act as an effective SARS-CoV-2 RNA-chain terminator, stopping its RNA reproduction [70].

**Figure 6:**
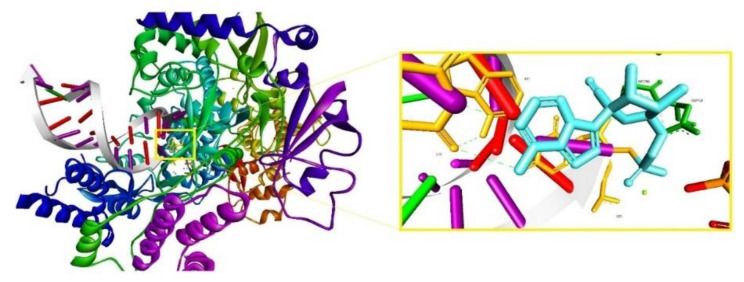
The nsp12-nsp7-nsp8 complex bound to the template-primer RNA and triphosphate form of RDV, Protein Data Base (PDB) ID: 7BV2 (left); RDV covalently bound to U20; hydrogen bonds are depicted as light green dotted lines, amino acid residues in dark green, and nucleotides in orange.

Following the same research direction, to evaluate the mechanism of action of RDV, a study published the 3D cryo-EM structure of SARS-CoV-2 RdRp in the apo form at 2.8 Å resolution and in complex with a template-primer RNA (50-base long) and RDV at 2.5 Å resolution [69]. According to this study, the characterized complex structure revealed that the partial double-stranded RNA template was inserted into the central channel of the RdRp, where RDV is covalently incorporated into the primer strand at the first replicated base pair, inhibiting the RdRp via non-obligated RNA chain termination. The complex structure is depicted in Figure 6 and is available in the RCSB Protein Data Base under the ID 7BV2 [69]. According to the authors, the structure comparison and sequence alignment analysis carried out on the obtained complex suggest that RNA recognition and RDV inhibition of RdRp is highly preserved in various RNA viruses, providing a basis for broad spectrum nucleotide analog antiviral-based drug design [69].

At the same time, in line with the reported findings related to RDV’s antiviral mechanism of action described by computational methods, several studies aimed to validate the presumed antiviral effect of RDV on SARS-CoV-2 through biological assessment.

Considerable proof has been gathered lately supporting the effectiveness of RDV against SARS-CoV-2 virus: (i) in vitro, Wang et al. proved that RDV efficiently inhibited the viral infection at low-micromolar concentrations in two different cell lines (Vero E6 and Huh-7) with a notable half-maximal effective concentration (EC50) value of 0.77 μM [56,61]; (ii) in vivo studies on animal models of SARS-CoV and MERS-CoV confirmed the RDV antiviral potential by reducing clinical symptoms of the infection [31]; and (iii) in clinical efficacy trials of RDV on severe COVID-19 patients, an improved clinical outcome was noticed, but several adverse reactions observed in the RDV-treated group were also reported [71,72].

Phase I, phase II, and phase III clinical trials on healthy volunteers and patients with Ebola virus infection asserted the pharmacokinetic properties and a safety profile of this compound (high human tolerance, no cytotoxicity, hepato- or renal toxicities, and no/reduced severe adverse effects were detected) [31].

Currently, RDV is tested in multiple ongoing phase 3 clinical trials for COVID-19 treatment (NCT04252664, NCT04257656, NCT04292730, NCT04292899, NCT04280705, Solidarity trial (WHO); DisCoVeRy trial (INSERM) in Belgium, and so on) and The European Medicine Agency (EMA) adopted Article 83, which provides recommendations on compassionate use of RDV for COVID-19 treatment in the European Union [73].

The manufacturer of Remdesivir, Gilead Sciences, Inc., sponsored two randomized, open-label, multi-center phase 3 clinical trials (also known as SIMPLE studies) that were developed in countries with a large number of cases: (1) SIMPLE trial 1 was developed on severe COVID-19 patients in order to assess the effectiveness and the safety of a 5-day versus 10-day treatment with RDV (first dose—200 mg/day intravenously administration, followed by 100 mg/day for 4 or 9 days, respectively) in addition to standard care, and (2) SIMPLE trial 2 performed on patients with moderate COVID-19 symptomatology aiming to evaluate the effectiveness and the safety of a 5-day or 10-day treatment with RDV versus standard care—the results will be available by the end of May [57].

On 29 April 2020, Gilead Sciences, Inc. provided in a press release the preliminary results obtained within SIMPLE trial 1 on severe COVID-19 patients, as follows: (i) a similar clinical improved outcome after a 5-day or 10-day treatment with RDV; (ii) an earlier start of RDV treatment (within 10 days of symptoms onset) reduces the hospitalization duration; and (iii) a high tolerance of RDV treatment (in both experimental settings: 5-day or 10-day treatment) in most patients, but some adverse effects occasionally occurred, such as nausea (10% of the patients), acute respiratory failure (a higher percentage in the 10-day group: 10.7% versus 6%), elevated liver enzymes (ALT) values (7.3% of the patients), and stopping the treatment in 3% of the patients owing to liver damage (high enzymes values) [57].

On 1 June 2020, Gilead Sciences, Inc. announced the results obtained from SIMPLE trial 2, as follows: (i) the 5-day treatment with RDV led to a significant improved clinical status in patients with moderate COVID-19 symptomatology at day 11 as compared with standard care group (in 65% of patients); and (ii) in terms of safety and adverse effects noticed, RDV treatment was well tolerated and the most common adverse effects observed were nausea (10% of the patients), diarrhea (5%), and headache (5% of the patients), and no deaths were recorded as compared with the standard care group that enlisted four deaths [74]. In addition, since 7 May 2020, Remdesivir (Veklury^®^) was approved in Japan as a treatment for patients with severe COVID-19 pathology [75].

Positive results regarding the effectiveness of RDV as a treatment for COVID-19 patients were also achieved in the Adaptive COVID-19 Treatment Trial (ACTT) financed by the National Institute of Allergy and Infectious Diseases (NIAID—part of the National Institutes of Health), a clinical trial that included 1063 patients: (i) patients treated with RDV (10 days—first day 200 mg/day intravenously, followed by 100 mg/day for 9 days) recovered faster (31%) as compared with patients that received placebo; in addition, the mortality rate was lower in the RDV-treated group compared with placebo (8% versus 11.6%, respectively) [57]. The preliminary report results of the ACTT study were published by Beigel et al. in The New England Journal of Medicine on 22 May 2020 [76]. On the basis of these preliminary promising data, European Medicine Agency (EMA) initiated the “rolling review” for RDV, resulting in the acceleration of the assessment process of RDV for marketing authorization [77], while the FDA (Food and Drug Administration) authorized RDV for emergency use as a treatment for hospitalized COVID-19 patients [78].

These latest data regarding RDV efficacy against SARS-CoV-2 infection are stimulating, although the gaps concerning its safety profile are rather wide at present, and the forthcoming results from the ongoing clinical trials are essential to fill them. On the basis of these considerations, it is recommended that, during the treatment, the physicians should be well-aware of the considerable number of factors/conditions (mainly in the case of severely ill patients with comorbidities—diabetes, cardiovascular pathology, and aged people) that might interfere with this compound and lead to adverse events. To prevent or to minimize any kind of undesired effects that might aggravate patients’ status, some key information about RDV pharmacokinetics, pharmacodynamics, and drug-drug interactions should be reviewed, data that will be provided in the following paragraphs.

According to the summary on compassionate use of RDV, this drug is formulated in two pharmaceutical forms (a solution—5 mg/mL and a lyophilized formulation—100 mg RDV powder) and is recommended to be administered intravenously (30–120 min) after reconstitution in 0.9% saline or 5% glucose solutions, with the therapeutic dosage being as follows: 200 mg on day 1 and 100 mg/day for the following 9 days. The recommended formulation of RDV for compassionate use is the lyophilized powder that must be reconstituted prior to use and administered intravenously, as stated above [73,79].

RDV is conditioned as a prodrug of the nucleoside analog GS-441524 and displays the following pharmacokinetic profile: 100% absorption after iv. administration (after oral administration RDV suffers an almost complete first-pass hepatic clearance), distribution as a free fraction in a proportion of 12.1% (moderate affinity for plasmatic proteins binding), and t1/2 (plasmatic half-life) of approximatively 1 h. Given its nucleotide analog structural features and high lipophilicity compared with that of the active form (Figure 5), RDV undergoes intense hepatic metabolization by cytochrome P450 enzymes (CYP2C8, CYP2D6, and CYP3A4) and is predominantly excreted in the urine as more hydrosoluble metabolites. The potential for interactions with other drugs is considered to be low for RDV (inhibitor of CYP3A4 and OATPs hepatic uptake transporter, according to several in vitro studies; no in vivo studies were performed) but is currently unknown for its metabolites.

In terms of the safety profile, it can be stated that this aspect is incompletely characterized, although several data were provided by the studies conducted by the manufacturer regarding the possible side effects (single doses ranging from 3 to 225 mg; multiple doses of 150 mg/daily for 7 to 14 days), such as phlebitis, constipation, headache, ecchymosis, nausea, pain in the extremities, as well as an increase of the hepatic enzymes values. The augmented values of hepatic transaminases are the only adverse effects directly associated with RDV according to manufacturer data; hypersensitivity reactions and renal events are also recommended to be monitored [79].

Nevertheless, considering that RDV as well as its active metabolite are both ATP competitors, potential future side effects may emerge owing to the fact that multiple kinase (which all have ATP binding sites) functions could be altered by possible RDV inhibition.

In addition to the side effects mentioned in the summary on compassionate use of RDV, a cohort study conducted by Grein and collaborators on severe hospitalized COVID-19 patients treated with RDV for 10 days (the dosage recommended for compassionate use: 200 mg on day 1 and 100 mg/day on day 2 to day 10) and supportive care reported other adverse effects, such as increased hepatic enzymes (most common), diarrhea, rash, renal disturbance, hypotension, acute kidney injury, atrial fibrillation, multiple-organ dysfunction syndrome, hypernatremia, deep-vein thrombosis, acute respiratory distress syndrome, pneumothorax, hematuria, delirium, septic shock, and pyrexia (most of these effects were noticed in patients on invasive ventilation). Taking into consideration the limitations of the study (small cohort size, a relatively short duration of the follow-up, lack of a randomized control group) and the paucity of information regarding COVID-19, it is questionable if these side effects are directly related to RDV administration or are in fact consequences/complications determined by the SARS-CoV-2 infection [71].

One of the randomized, double-blind, placebo-controlled, multicentre clinical trials developed in China (NCT04257656) in order to verify RDV efficacy as a COVID-19 treatment (the dosage recommended for compassionate use was applied: 200 mg on day 1 followed by 100 mg/daily from day 2 to day 10 co-administered with lopinavir-ritonavir/interferons/corticosteroids) indicated an improved outcome in patients treated with RDV, with a decreased duration of invasive mechanical ventilation; however, these benefits were not statistically significant. Several adverse effects were recorded within the RDV-treated group and were classified as follows: most common—constipation, hypoalbuminemia, hypokalemia, thrombocytopenia, anemia, and an augmented total bilirubin; common—increased blood glucose, rash, augmented blood lipids, high white blood cell count, hyperlipidemia, increased neutrophils number, high blood urea nitrogen, augmented aspartate aminotransferase, nausea, diarrhea, reduced sodium level, and increased serum potassium, as well as rare severe adverse events as respiratory failure, acute respiratory distress syndrome, and cardiopulmonary failure. Even though a plethora of side-effects in the RDV-treated group were reported, these reactions could not be directly correlated to RDV, and the conclusion of the authors in terms of RDV safety profile was that this drug “was adequately tolerated and no new safety concerns were identified”. Moreover, the average proportion of severe adverse events was lower in the RDV-treated group compared with the placebo group [72].

The in vitro metabolism studies (for the identification of drug interactions) for RDV revealed an inhibitory effect on CYP3A4 enzyme; as this enzyme catalyzes multiple drug metabolism pathways, it is important to determine if RDV could interfere with these drugs and initiate different side effects. An ample study in this direction was conducted by the Liverpool Drug Interactions Group (University of Liverpool), and revealed the following findings: (i) co-administration of RDV with drugs from different classes as analgesics (metamizole), antibacterials (rifabutin), anticonvulsants (eslicarbazepine, oxcarbazepine, rufinamide), anti-hypertensives for pulmonary hypertension (bosentan), and steroids (betamethasone, dexamethasone) can lead to potential interaction that might require an adjustment of the RDV dose or a close patient monitoring; and (ii) co-administration of RDV with drugs from the classes of antibacterials (rifampicin, rifapentine), anticonvulsants (carbamazepine, phenobarbital, phenytoin, primidone), and antidepressants (St John’s wort) is not recommended.

Key information is represented by the lack of clinically significant interaction between RDV and other classes of drugs used as comedication for COVID-19 patients as analgesics, antiarrhythmics, antibacterials, anticoagulants, anti-platelets and fibrinolytics, antidiabetics, antifungals, anti-hypertensives (ACE inhibitors, angiotensin antagonists, diuretics, other agents), neuroleptics/antipsychotics, other antivirals for COVID-19 treatment, anxiolytics/hypnotics/sedatives, beta blockers, bronchodilators, calcium channel blockers, contraceptives, gastrointestinal agents, anti-emetics, hormone replacement therapy, immunosuppressants, inotropes and vasopressors, lipid lowering agents, and steroids [80].

In view of all the data stated above about RDV, several recommendations and cautions are suggested:

- The duration of RDV treatment should be settled at 5 days (200 mg day 1 followed by 100 mg/daily for the following 4 days) in severe COVID-19 cases—on the basis of the results obtained within SIMPLE trial 1, this measure represents a major step in reducing the risk of drug–drug interactions/side-effects/complications/aggravation of patient status;
- As the RDV directly-linked adverse effect is hepatic injury, a possible preventive approach would consist of the co-administration of hepatoprotective medication (for example, essentials phospholipids or albumin solution—it should be a patient-dependent decision after analyzing the risk/benefit ratio) during the RDV treatment (in severely ill COVID-19 patients, liver dysfunction was reported, but until now, it is unclear what causes it—the medication administered or the SARS-CoV-2 infection, thus liver protection would do no harm);
- As RDV is predominantly eliminated through urine (mostly as metabolites), the renal function should be monitored during the treatment and, in the case of renal impairment, the treatment should be stopped
- Co-administration of other anti-COVID-19 drugs (lopinavir/ritonavir, chloroquine, hydroxychloroquine, interferon) is possible because no drug–drug interactions were reported; surveillance is recommended
- Co-administration of antidiabetics and cardiovascular medication (anti-hypertensives, anticoagulants, beta blockers, and so on) is possible because no drug–drug interactions were reported; surveillance is recommended
- Co-administration of RDV with other hepatotoxic drugs is not recommended
- To remedy/to prevent the mild adverse reactions reported in the clinical trials, such as nausea, diarrhea, hyperlipidemia, and so on, symptomatic medication could be administered during RDV treatment because no interactions were described between these classes of drugs and RDV; strict surveillance is recommended
- To follow the updates on other clinical trials that tested RDV.

Every medical decision regarding the treatment of COVID-19 patients should be taken after a thorough analysis of the risk/benefit ratio and all adverse events should be reported in order to fill the gaps in the RDV toxicological profile.

### 3.2. Chloroquine and Hydroxychloroquine

**Figure 7:**
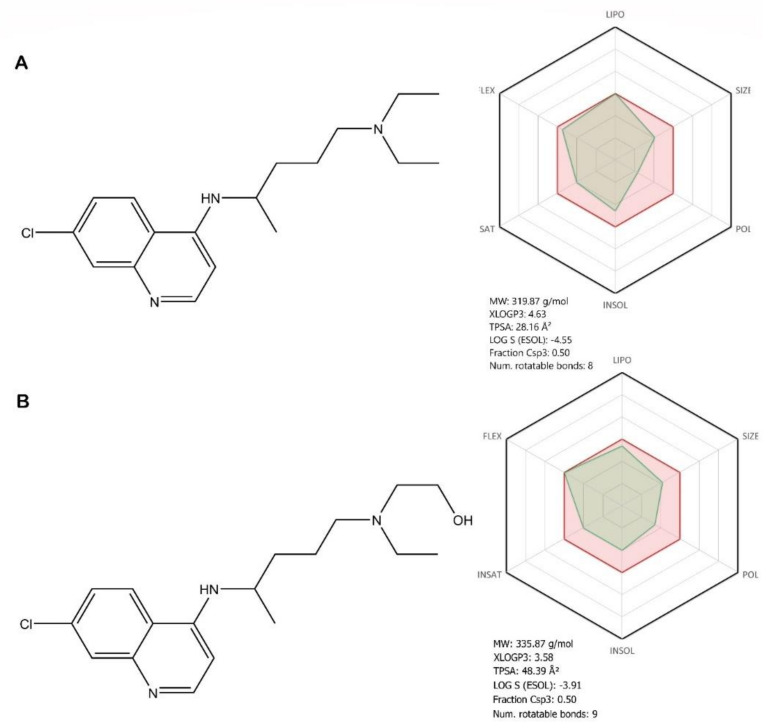
Chemical structure of chloroquine (**A**) and hydroxychloroquine (**B**) ADME profile was achieved using the free web tool SwissADME; the red highlighted area represents the suitable physicochemical space for oral bioavailability, covering value intervals for the following: LIPO (lipophility): −0.7 < XLOGP3 < +5.0, SIZE: 150 g/mol < MV < 500 g/mol, POLAR (polarity): 20Å^2^ < TPSA < 130Å^2^, INSOLU (insolubility): 0 < Log S (ESOL) < 6, INSATU (insaturation): 0.25 < Fraction Csp3 < 1, FLEX (flexibility): 0 < Num. rotatable bonds < 9, whereas the overlapped green highlighted area shows the calculated ADME profile for the molecule [63].

Chloroquine (CQ—Figure 7A, see brief description in Table 2), a 70-year-old antimalaria drug, currently one of the agents for amoebiasis and other protozoal diseases and antimalarials associated with irreversible retinal damage and life-threatening and fatal cardiomyopathy, has been recently reported as a potential broad-spectrum antiviral drug [61,81].

Hydroxychloroquine (HCQ—Figure 7B, see brief description in Table 2) is a chloroquine analogue [82], one of the antimalarials and other anti-inflammatory and antirheumatic agents associated with ocular toxicity and cardiomyopathy, is currently recommended in the treatment of immune diseases, such as systemic lupus erythematosus and rheumatoid arthritis [83]. HCQ has been used for the last 30 years to treat intracellular bacterium *Coxiella burnetii*, which produces the Q fever, being the only effective agent that kills intracellular pathogens. Another important therapeutic activity is against the intracellular bacterium *Tropheryma wippley* [84].

Structurally, both compounds are 7-chloro-quinoline derivatives, with a novaldiamine substituent in the fourth position, where HCQ has an additional hydroxyl group grafted at the end of the chain. In terms of bioavailability, the additional hydroxyl group, according to the absorption, distribution, metabolism, and excretion (ADME) profile of HCQ, leads, as expected, to an improvement in hydrosolubility, flexibility, and polarity, as well as to a decrease in lipophilicity compared with CQ (Figure 7). These differences in the ADME profiles of the two molecules, owing to the presence of the hydroxyl group, can lead to different pharmacological behaviors, in terms of therapeutic efficacy and outcome, but also in the occurrence of toxic effects. These pharmacokinetic aspects will be further discussed.

The reposition of CQ and HCQ as antiviral candidates for COVID-19 treatment was based on several in vitro and in vivo studies that reported their therapeutic effect against several coronaviruses, such as human OC43, SARS-CoV, and MERS-CoV [81,85]. The molecular mechanism of action of CQ and HCQ has not been fully elucidated yet [82].

Previous studies have explored the mechanism of the antiviral action of CQ against SARS-CoV. The authors concluded that a possible mode of action of the drug, in post-infection treatment, would be the increase of the endosomal pH value, owing to the presence of the three nitrogen atoms within the CQ molecule, which give its basic properties, leading to abrogation of virus-endosome fusion [86,87]. These findings also suggest that pre-infection treatment with CQ is responsible for cell surface expression of under-glycosylated ACE2, leading to a decrease in the viral spike protein–cell receptor affinity [86]. In line with these reports, a recent study also showed that CQ/HCQ induced pH elevation, within acidic intracellular organelles, such as early endosomes (EEs) and endolysosomes (ELs), and also caused the disruption of SARS-CoV-2 transport between EE and EL [83]—a phase that seems to be required in viral genome release, in SARS-like coronavirus infections [88].

At the same time, regarding the molecular mechanism of action of CQ/HCQ, the possibility to find a potential target, until experimental validation, was the subject of in silico determinations. Wu et al. screened two compound libraries (ZINC and a natural compound library of their own) against 19 SARS-CoV-2 protein targets. Among the results, the authors showed that chloroquine can target nonstructural proteins such as Nsp3b, exhibiting suitable docking scores [89].

In terms of anti-inflammatory and immunomodulatory activities, a number of mechanisms have been proposed, for both CQ and HCQ, which involve the following: decreased cytokine production, suppression of the immune effector cells and platelet function, protection of the cell surface from external disorders, competitive binding to nucleic acid ligands or toll-like receptors (TLRs), interference with lysosomal function, reduction of lysosomal enzyme leakage, and interference with endosomal NADPH oxidase (NOX) [90].

Depending on their activity against SARS-CoV-2, the possible mechanisms of action can be divided into two main categories: (1) inhibition of viral enzymes/processes (viral DNA and/or RNA polymerase), glycosylation of viral proteins, assemblage of virus, new virus particle transport, and virus release; and (2) ACE2 cellular receptor inhibition, acidification at the surface of the cell membrane thus inhibiting virus fusion, and immunomodulation of cytokine release [90].

The promising in vitro results of CQ and HCQ against CoVs led to an early clinical interest in the use of these two compounds as therapy for COVID-19 and multiple clinical trials (over 50, most of them evaluating the HCQ effects [51], were set in motion [91]). The data obtained from the clinical studies (final results or pre-print texts) present methodological flaws [91] and are inconclusive: (i) an improved clinical outcome was observed in HCQ-treated group, but was not statistically relevant [92]; (ii) coadministration of HCQ with azithromycin determined the decrease of viral load in COVID-19 patients [93]; (iii) CQ inhibited pneumonia exacerbation and shortened the infection course (improved lung imaging and increased viral clearance) [94]; (iv) HCQ proved to be better in terms of efficacy compared with CQ [95,96]; and (v) HCQ apparently offered no protection against SARS-CoV-2 infection (results of a large healthcare Israelian database analysis) [97]. The association of HCQ and azithromycin for the treatment COVID-19 patients was based on several premises: azithromycin proved in vitro activity against Ebola and Zika viruses, as well as a preventive effect against severe respiratory tract infections to patients presenting viral infection [93], but further studies confirmed the efficacy of HCQ and azithromycin combination [98].

CQ and HCQ are also evaluated in three ample clinical trials; that is, the SOLIDARITY trial sponsored by WHO that evaluates these compounds as possible therapy against SARS-CoV-2 infection; the HERO-HCQ, a study sponsored by the National Institutes of Health (NIH) that assesses their preventive potential; and the DisCoVeRy trial, launched by INSERM (French Institut National de la Santé Et de la Recherche Médicale) [99,100]. Even though the two compounds share similar chemical structures, it has been reported that HCQ presents some therapeutic benefits when compared with CQ such as a lower toxicity in animals [83]. It is important to note that both CQ and HCQ interfere with the cytochrome P450 isoenzymes and drug transporters: CYP2C8, CYP2D6, CYP3A4, P-gp being primarily metabolized by CYP2C8 and CYP3A4, and known inhibitors of the drug transporter P-glycoprotein (P-gp), thus explaining the interactions (increasing/decreasing) with associated antiviral drugs. Both CQ and HCQ show favorable pharmacokinetic properties: efficient oral absorption and tissue distribution patterns, leading to high concentrations in the liver, spleen, kidney, and lungs for CQ and the bone marrow, liver, kidneys, lungs, adrenal gland, and pituitary gland for HCQ. It should be noted that melanin-containing cells bind strongly to the chloroquine process, explaining the retinal toxicity of both compounds [82,90].

Moreover, given the fact that CQ and HCQ are both quinine/quinidine analogs, one major concern in the therapeutically use of these drugs is related to the QTc interval prolongation and the increased incidence of ventricular arrhythmias [101].

From a toxicological point of view, both substances show significant adverse reactions and drug–drug interactions, as follows: most of the antiarrhythmics (amiodarone, bepridil, flecainide, mexiletine) are not recommended to be coadministered with CQ and HCQ owing to the increased risk of QT development; several antibacterials (rifampicin, rifapentine) and anticonvulsants (phenobarbital, phenytoin, primidone, carbamazepine) are also not recommended as co-medication.

The most common adverse effects associated with CQ and HCQ use are gastrointestinal disorders, hypoglycemia, and QT interval prolongation (after short term treatment), as well as cardiomyopathy and retinal toxicity (after long-term treatment) [102]. Several concerns were raised regarding the safety profile of HCQ administered for COVID-19 treatment (the doses recommended in several guidelines for COVID-19 treatment—800 mg/day on day 1, followed by 400 mg/day for 4 days —are higher as compared with those administered conventionally for chronic therapies) in terms of cardiac toxicity and retinopathy [101,103]. Moreover, a retrospective study conducted on 95 hospitalized COVID-19 patients treated with CQ showed that 23% of the patients presented QT interval prolongation [104]. A case report stated that CQ treatment induced Torsade de Pointes in a COVID-19 female patient [105].

Coadministration of CQ and HCQ with the other investigational anti-COVID-19 agents (LPV/r, atazanavir) should be performed under strict monitoring, as LPV/r increases their concentrations and, subsequently, the risk for adverse effects. Moreover, when the coadministration is decided, ECG (electrocardiogram) monitoring is required, and the treatment period should be as short as possible. No interactions were reported so far between RDV and CQ or HCQ; therefore, their coadministration can be considered safe, at least until further notice [80].

The current preclinical and clinical data regarding the use of CQ and HCQ in SARS-CoV-2 infection are not considered robust [102], and EMA did not approve chloroquine for standard use in COVID-19 pathology and restricted its use exclusively in clinical trials or through national emergency use programs [101,106].

At present, there are more than 200 ongoing clinical studies enlisted regarding the use of CQ/HCQ alone or in combination with macrolides (azithromycin or clarithromycin) as treatment or as pre- or post-exposure prophylaxis in COVID-19 patients. Although positive results were obtained in small size clinical studies, the recent data collected from larger size studies indicate that these drugs have no significant benefit in clinical outcome; moreover, their association with macrolides might augment the risk of cardiac adverse effects (arrhythmia) and mortality of hospitalized patients [98].

In addition, the latest update provided by WHO on 17 June 2020 regarding the assessment of hydroxychloroquine as a treatment for COVID-19 infection notifies that this part of the SOLIDARITY trial was suspended based on the evidence gathered according to which hydroxychloroquine had no impact on decreasing the mortality of hospitalized COVID-19 patients as compared with standard care. Similar results were furnished by the United Kingdom’s Recovery Trial, DisCoVeRy trial, and a Cochrane review, which also stopped their study [107].

### 3.3. Lopinavir/Ritonavir

**Figure 8:**
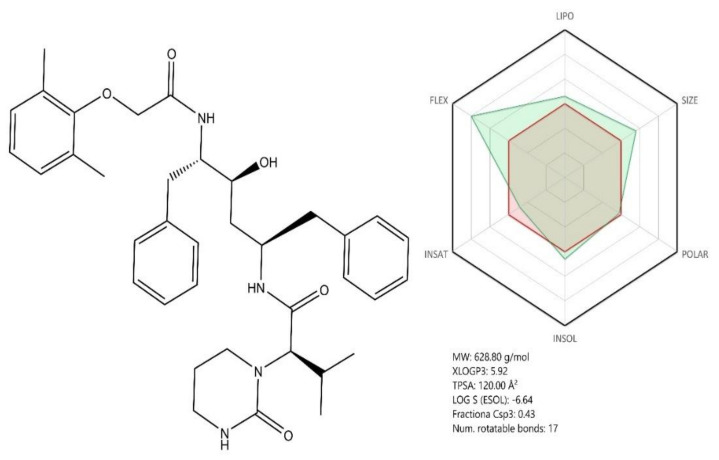
Chemical structure of Lopinavir; ADME profile was achieved using the free web tool SwissADME; the red highlighted area represents the suitable physicochemical space for oral bioavailability covering value intervals for the following: LIPO (lipophility): −0.7 < XLOGP3 < +5.0, SIZE: 150 g/mol < MV < 500 g/mol, POLAR (polarity): 20Å^2^ < TPSA < 130Å^2^, INSOLU (insolubility): 0 < Log S (ESOL) < 6, INSATU (insaturation): 0.25 < Fraction Csp3 < 1, FLEX (flexibility): 0 < Num. rotatable bonds < 9, whereas the overlapped green highlighted area shows the calculated ADME profile for the molecule [63].

**Figure 9:**
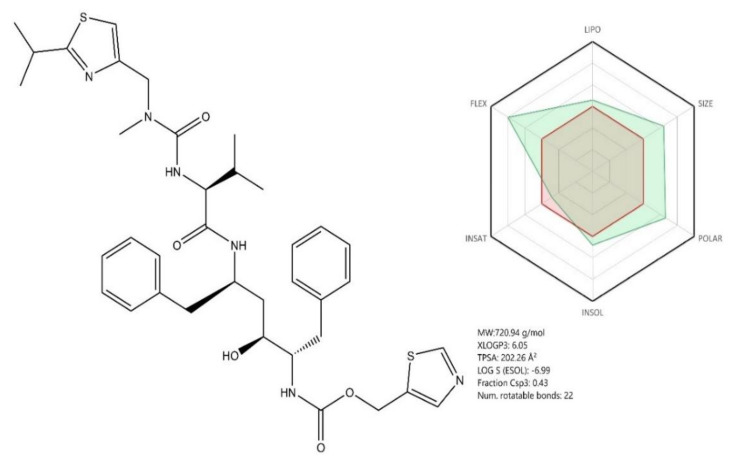
Chemical structure of Ritonavir; ADME profile was achieved using the free web tool SwissADME; the red highlighted area represents the suitable physicochemical space for oral bioavailability covering value intervals for the following: LIPO (lipophility): −0.7 < XLOGP3 < +5.0, SIZE: 150 g/mol < MV < 500 g/mol, POLAR (polarity): 20Å^2^ < TPSA < 130Å^2^, INSOLU (insolubility): 0 < Log S (ESOL) < 6, INSATU (insaturation): 0.25 < Fraction Csp3 < 1, FLEX (flexibility): 0 < Num. rotatable bonds < 9, whereas the overlapped green highlighted area shows the calculated ADME profile for the molecule [63].

Lopinavir and ritonavir (LPV/r) (Figure 8, see brief description in Table 2) are two antiviral drugs acting as viral protease inhibitors, currently used against HIV (human immunodeficiency virus) infection [108]. Structurally, the two compounds are 1,6-diphenyl-4-hydroxy-2,5-diaminohexane derivatives whose amino groups contain side chains with various substituted cyclic moieties such as phenoxy/3,5-diazinan (Lopinavir) or 1,3-thiazole (Figure 8). Given their molecular structure, both compounds share similar pharmacokinetic profiles based on their calculated ADME profile (Figure 8 and Figure 9). Previous studies showed that the combination of the two compounds had a beneficial effect against CoVs infections such as SARS-CoV, which triggered the investigation of their potential antiviral effect against SARS-CoV-2 as well [109].

CoVs encode specific proteases such as papain—like protease (PLpro) and main protease (Mpro or 3CLpro) [48,108]. Both enzymes are involved in the control of the CoVs’ gene expression and replication [108] by inducing the proteolysis of viral polyproteins into functional and individual units [48] consisting of spike proteins, membrane proteins, envelope proteins, nucleoproteins, replicases, and other polymerases [110]. Mpro, a highly conserved homodimer among Coronaviridae members such as SARS-CoV and MERS-CoV, has emerged as a potential target for lopinavir/ritonavir owing to its role in self and polyprotein maturation [108]. A previous study assessed the ability of several antivirals to act as inhibitors of the SARS-CoV specific viral protease Mpro, by means of molecular docking and molecular dynamic simulations. The conclusion was that the molecules with the best scores for potential viral protease inhibition were lopinavir and ritonavir [111]. In accordance with previous studies, to investigate a possible mechanism for the antiviral activity of LPV/r, Muralidharan et al. performed molecular docking and molecular dynamics simulations to explore the affinity of these antiretroviral drugs to the SARS-CoV-2 protease, and their results showed a stronger binding energy of the two drugs combined compared with that exerted by each drug separately [108]. Their study also revealed that Lopinavir was able to interact with the viral protease predominantly through hydrophobic interactions, while in the Ritonavir–protease complexes, both hydrogen bonding and hydrophobic interactions were deemed as key features for enzyme inhibition [108].

However, the LPV/r combination in the treatment of COVID-19 is not justified or, at least, needs further investigations. Cao B and co-workers conducted a randomized, controlled, open-label trial in adult hospitalized patients with COVID-19 to test the efficacy and safety of the oral lopinavir (400 mg) associated with ritonavir (200 mg) in SARS-CoV-2 infection. At the end of their study, there was no evidence that LPV/r combination exerted any significant antiviral effect against SARS-CoV-2, as the viral RNA was still detected in 40.7% of the patients belonging to the LPV/r group [31,112]. Moreover, European Society of Intensive Care Medicine (ESICM) does not currently recommend LPV/r for routine use [31,113].

The pharmacokinetic profile of LPV/r consists of the following parameters: (1) a good absorption rate after oral administration, (2) lopinavir presents a high affinity for serum proteins (98–99% bound to serum proteins, mainly of alpha-1-acid glycoprotein (AAG)), (3) hepatic biotransformation of lopinavir is almost exclusively by isozyme CYP3A (ritonavir is a strong inhibitor of CYP3A, increasing the levels of lopinavir), and (4) elimination in urine and feces both untransformed and metabolized. Taking into consideration that LPV/r (Kaletra) was approved as anti-HIV therapy for almost 20 years ago (2001), a long list of adverse effects was ascribed to this product, classified as follows: (i) very common: upper respiratory tract infection, diarrhea, nausea; (ii) common: lower respiratory tract infection, skin infections (including cellulitis, folliculitis, and furuncle), anemia, leucopenia, neutropenia, lymphadenopathy, urticaria, angioedema, blood glucose disorders including diabetes mellitus, hypertriglyceridemia, hypercholesterolemia, weight decrease, decreased appetite, anxiety, headache (migraine), insomnia, dizziness, hypertension, pancreatitis, abdominal pain, hepatitis (with increased AST (aspartate aminotransferase), ALT, and GGT (Gamma-glutamyl transferase) values), skin disorders, myalgia, and erectile dysfunction, among others; (iii) uncommon: immune reconstitution inflammatory syndrome, hypogonadism, weight increase, abnormal dreams, cerebrovascular accident, convulsion, dysgeusia, ageusia, tremor, visual impairment, tinnitus, deep vein thrombosis, gastrointestinal hemorrhage, gastrointestinal ulcer, jaundice, hepatic steatosis, alopecia, rhabdomyolysis, osteonecrosis, and nephritis, among others; and (iv) rare: Stevens–Johnson syndrome and erythema multiforme [114].

As both lopinavir and ritonavir are CYP3A enzyme inhibitors, multiple drug–drug interactions were described, thus increased attention is recommended when this combination is co-administered with other substances metabolized by the same enzyme. Among the long list of interactions (for the detailed list, visit [80]), we will present several contraindicated drugs to be co-administered with LPV/r in the COVID-19 actual context: dextropropoxyphene, amiodarone, bepril, dofetilide, rifampicin, apixaban, clopidogrel, ticagrelor, St John’s wort, aliskiren, ivabradine, lercanidipine, ranolazine, sildenafil, quetiapine, antivirals such as hepatitis C virus (HCV) DDAs (direct-acting antiviral agents), midazolam, triazolam, cisapride, domperidone, sirolimus, lovastatin, simvastatin, budesonide, fluticasone, mometasone, and triamcinolone [114].

In light of the toxicological profile of LPV/r and its high potential for drug–drug interactions, China National Center for Adverse Drug Reaction Monitoring collected and analyzed data from hospitals concerning medication safety for COVID-19 patients; their report included the following findings: 179 patients (82.5%) developed adverse effects after the lopinavir/ritonavir treatment and 37 patients (17.1%) after chloroquine use. The adverse effects related to LPV/r use consisted of the following: hypertriglyceridemia, hypercholesterolemia, gastrointestinal reactions (diarrhea), liver injury, and rush [115].

In the opinion of the Infectious Diseases Society of America (IDSA), the published clinical data concerning LPV/r efficacy as COVID-19 treatment are not sufficient to recommend this medicine as treatment, only in a clinical trial context [116].

## 4. Potential Promising Antiviral Agents

Besides the interest in the repurposed drugs included in the interim guidelines for COVID-19 treatment, a great interest was attracted globally by the new anti-SARS-CoV-2 agents. A considerable number of articles was dedicated to this subject (more than 10,000 articles found in PubMed database) and, in the next section, the most studied of these compounds in the actual context will be briefly discussed.

### 4.1. Oseltamivir

**Figure 10:**
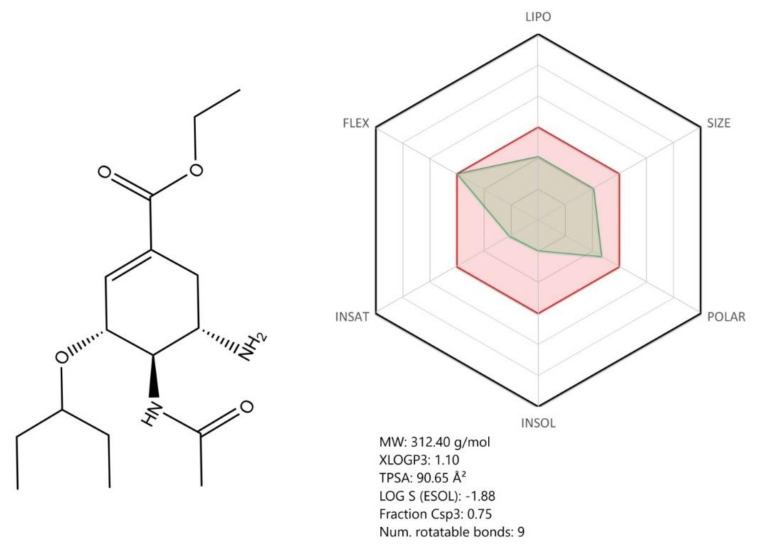
Chemical structure of oseltamivir ADME profile was achieved using the free web tool SwissADME; the red highlighted area represents the suitable physicochemical space for oral bioavailability covering value intervals for the following: LIPO (lipophility): −0.7 < XLOGP3 < +5.0, SIZE: 150 g/mol < MV < 500 g/mol, POLAR (polarity): 20Å^2^ < TPSA < 130Å^2^, INSOLU (insolubility): 0 < Log S (ESOL) < 6, INSATU (insaturation): 0.25 < Fraction Csp3 < 1, FLEX (flexibility): 0 < Num. rotatable bonds < 9, whereas the overlapped green highlighted area shows the calculated ADME profile for the molecule [63].

Oseltamivir (Figure 10), a neuraminidase inhibitor, has been specifically developed for treating the influenza virus infection [108]. Being structurally designed based on DANA (2,3-dehydro-2-deoxy-N-acetylneuraminic acid), oseltamivir contains a cyclohexene ring with two different substituents compared with DANA; a C4 amino group and a bulky hydrophobic pentyl ether side chain [121]. Oseltamivir is administered as a phosphate prodrug, which is converted by hepatic esterases to the active carboxylate form [122].

On the basis of its ADME profile, oseltamivir shows overall good bioavailability for a drug intended for oral administration. It was previously shown that oseltamivir has an 80% bioavailability upon oral administration and a limited potential for clinically relevant interactions with commonly co-administered drugs [122]. Given its structure and metabolic pathway, oseltamivir does not contain known toxicophore groups or precursors.

According to a recent study, oseltamivir forms ligand–enzyme complexes with the SARS-CoV-2 proteases via hydrogen and hydrophobic bonds, manifesting a synergistic activity with lopinavir and ritonavir. The authors concluded that the effect of the three drugs together (lopinavir, ritonavir, oseltamivir) against the protein is stronger than that of each drug individually, suggesting that this association might be helpful in the COVID-19 treatment [107]. Given these aspects, in the event of additional studies that may validate its antiviral activity on SARS-CoV-2, oseltamivir may be a safe candidate for clinical testing.

### 4.2. Ribavirin

**Figure 11:**
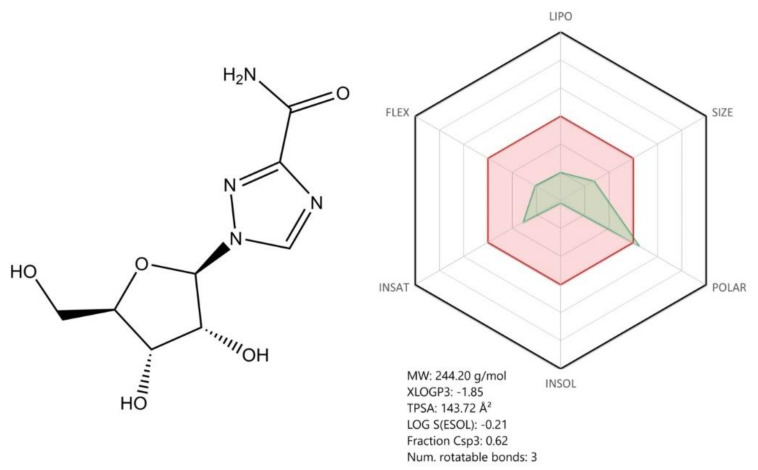
Chemical structure of ribavirin; ADME profile was achieved using the free web tool SwissADME; the red highlighted area represents the suitable physicochemical space for oral bioavailability covering value intervals for the following: LIPO (lipophility): −0.7 < XLOGP3 < +5.0, SIZE: 150 g/mol < MV < 500 g/mol, POLAR (polarity): 20Å^2^ < TPSA < 130Å^2^, INSOLU (insolubility): 0 < Log S (ESOL) < 6, INSATU (insaturation): 0.25 < Fraction Csp3 < 1, FLEX (flexibility): 0 < Num. rotatable bonds < 9, whereas the overlapped green highlighted area shows the calculated ADME profile for the molecule [63].

Ribavirin (Figure 11) is a nucleoside analog antiviral agent. Structurally, ribavirin was modified so that the nucleobase part was replaced by a 1,2,4-triazole ring with a carboxamide group in the third position. ADME profile wise, ribavirin is a highly soluble, highly polar rigid molecule, suited both for oral and iv administration. Thanks to its high hydrosolubility, its oral bioavailability reaches 50%, but can be increased when co administrated with a fatty meal [123].

In terms of its antiviral mechanism of action, after being metabolized to its triphosphate form, ribavirin competes with the physiological nucleoside (adenosine/guanosine) for the RdRp active site [124] and is incorporated in the RNA strand, but in some experimental conditions, does not terminate RNA chain elongation, but rather acts as a viral mutagenesis inducing agent [125]. The drug is currently used in the treatment of hepatitis C virus (HCV) infection [124].

Using docking experiments, a research team demonstrated that ribavirin and other FDA-approved antiviral drugs (galidesivir, remdesivir, tenofovir, sofosbuvir) are able to bind to SARS-CoV-2 RdRp, with binding energies comparable to those of native nucleotides, suggesting their potential use in COVID-19 therapy [126]. However, in vitro studies were conducted in order to explore the specific antiviral activity of Ribavirin against SARS-CoV-2; the results concluded that high concentrations of the drug are required to reduce the viral infection, as the EC_50_ value (109.50 μM) of ribavirin was higher than that of other antivirals such as remdesivir [61].

### 4.3. Arbidol Hydrochloride (Umifenovir)

**Figure 12:**
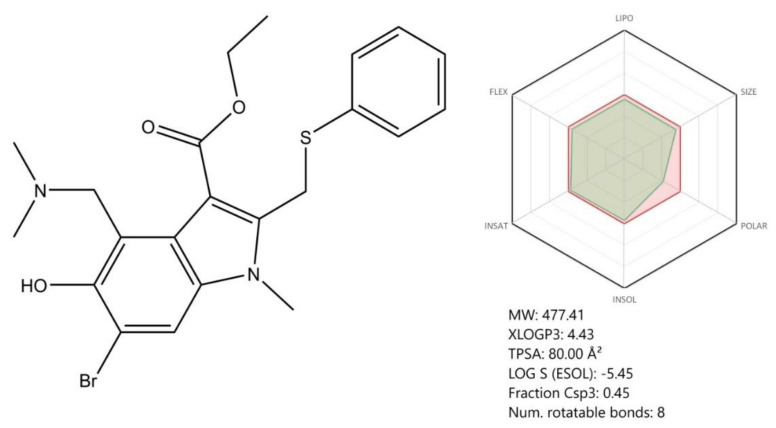
Chemical structure of Umifenovir; ADME profile was achieved using the free web tool SwissADME; the red highlighted area represents the suitable physicochemical space for oral bioavailability covering value intervals for the following: LIPO (lipophility): −0.7 < XLOGP3 < +5.0, SIZE: 150 g/mol < MV < 500 g/mol, POLAR (polarity): 20Å^2^ < TPSA < 130Å^2^, INSOLU (insolubility): 0 < Log S (ESOL) < 6, INSATU (insaturation): 0.25 < Fraction Csp3 < 1, FLEX (flexibility): 0 < Num. rotatable bonds < 9, whereas the overlapped green highlighted area shows the calculated ADME profile for the molecule [63].

Arbidol hydrochloride (Figure 12) is an indole-based derivative antiviral agent that acts as an influenza virus inhibitor and is clinically approved only in Russia and China. Its mechanism of action consists of binding hemagglutinin (HA), a major glycoprotein that is located on influenza virus surface and blocking the fusion of the viral membrane with the endosome. As SARS-CoV-2 also presents hemagglutinin (HA) on its surface, it was hypothesized that Arbidol could be efficient against SARS-CoV-2 [112,127,128]. At present, Arbidol is subjected to clinical trials both as a single agent and in combination with favipravir [112].

### 4.4. Favipiravir

Favipiravir, (6-fluoro-3-hydroxypyrazine-2-carboxamide), is a prodrug antiviral agent approved in Japan for the treatment of influenza. The compound is a nucleic acid purine base analog that acts primarily by inhibiting viral RdRp. Other mechanisms of action reported involve RNA induced lethal mutagenesis. Like other representatives of this class, favipiravir is metabolized to its active form, favipiravir-ribofuranosyl-5′-triphosphate, responsible for the pharmacological effect [129]. Favipiravir is metabolized by hepatic aldehyde oxidase in the cytosol (it is not metabolized by microsomal enzymes) and does not produce significant drug–drug interactions. Moreover, it does not affect the human DNA polymerases α, β, and γ subunits (at up to 100 µg/mL), thus being non-toxic [129].

A clinical trial (ChiCTR2000029600) carried out to evaluate the safety and efficacy of favipiravir for the treatment of SARS-Cov-2 conducted in Shenzhen, on 80 patients, concluded that, compared with lopinavir/ritonavir, favipiravir treatment reduced viral clearance time, and 91% of patients showed improved CT scans with few side effects. The drawback of this study was that it was not randomized double-blinded and placebo-controlled [130]. Another multicentered randomized clinical study (ChiCTR200030254) showed that favipiravir treatment increased the seven-day clinical recovery rate from 55.86% to 71.43%, in co-morbidity free COVID-19 patients. Moreover, favipiravir treatment decreased the time of fever reduction and cough relief within co-morbidity free/co-morbidity associated COVID-19 patients [131].

Favipiravir is currently approved in Russia for COVID-19 treatment, only in hospital settings [132].

### 4.5. Betulinic Acid

Natural compounds are highly investigated for treating a broad spectrum of diseases and those manifesting antiviral activity might be of great use as adjuvants in COVID-19 therapies. For instance, Khaerunnisa et al. investigated the ability of some natural compounds such as kaempferol, quercetin, oleuropein, curcumin, catechin, and others to serve as potential inhibitor candidates for the SARS-CoV-2 main protease (Mpro). According to their paper, the affinity of kaempferol to Mpro is higher than that of other natural compounds and comparable to some FDA-approved drugs such as nelfinavir and lopinavir [133].

**Figure 13:**
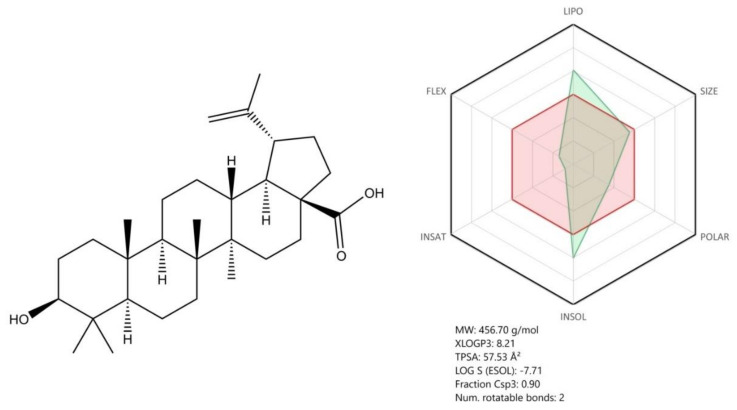
Chemical structure of betulinic acid ADME profile was achieved using the free web tool SwissADME; the red highlighted area represents the suitable physicochemical space for oral bioavailability covering value intervals for the following: LIPO (lipophility): −0.7 < XLOGP3 < +5.0, SIZE: 150 g/mol < MV < 500 g/mol, POLAR (polarity): 20Å^2^ < TPSA < 130Å^2^, INSOLU (insolubility): 0 < Log S (ESOL) < 6, INSATU (insaturation): 0.25 < Fraction Csp3 < 1, FLEX (flexibility): 0 < Num. rotatable bonds < 9, whereas the overlapped green highlighted area shows the calculated ADME profile for the molecule [63].

Betulinic acid (3β-hydroxy-lup-20(29)-en-28-oic acid—Figure 13) (BA) is a pentacyclic lupan triterpene usually isolated from birch trees, but present in many other botanical sources. BA exhibits important and diverse biological properties including antimalarial, anti-inflammatory, anticancer, and antiviral activity [134], which might become relevant within the novel SARS-CoV-2-induced infection therapy. In terms of BA’s antiviral effect, recent papers described its activity against several viruses [134].

As an anti-HIV-1 agent, it inhibits the replication and maturation of the virus by preventing the cleavage of the capsid–spacer peptide of the Gag protein [134], which is crucial to the structural alterations necessary for the formation of mature HIV-1 particles [135]. Through this mechanism of action, BA causes the host cell to release virions with no infective ability [134]. One of the hurdles for betulinic acid to release its antiviral potency is its poor water solubility, which persuaded scientists to synthesize more water-soluble BA derivatives and examine their biological activities [135]. Derivatives such as dihydrobetulinic acid, 3-alkylamido-3-deoxi-betulinic acid, or 3-O-(3-3-dimethylsuccinyl betulinic acid) were reported as potent antivirals. The last compound is involved in the assembly and/or budding of virions by blocking a late step of virus replication [135]. The pharmacological effect of a compound is strictly linked to its chemical structure; thus, according to recent findings, the most potent antiviral compounds were those with an ortho halogen substitution in the benzoic moiety of the dehydrobetulinic and betulin derivatives, and the simple benzoic or phthalic acids of dehydrobetulin [134]. Other derivatives such as Ω-undecanoic amides and ionic derivatives of betulinic acid conjugated with glycine intervene during the fusion of the virus to the cell membrane and cause the inhibition of HIV-1 protease activity, respectively.

In the above mentioned virtual screening study by Wu et al., out of their natural compound library, which was screened against the 19 SARS-CoV-2 target proteins, a similar pentacyclic triterpene, betulonal, emerged as a top possible RdRp and 3CLpro inhibitor [89].

Even though the most recent papers focus almost exclusively on its anti-HIV activity [134], BA showed promising results against other common viruses as well such as the herpes simplex virus (HSV). Phillips et al. reported that the ionic derivatives of betulinic acid displayed improved water solubility and a stronger antiviral activity against HSV type 2 compared with BA [135]. Moreover, in vitro studies on human lung carcinoma A549 cells revealed BA’s capacity to inhibit the proliferation of an influenza virus strain in a dose-dependent manner [47].

Previous investigations focused on testing the antiviral activity of lupane-type triterpenes such as BA on SARS coronaviruses. According to Wen C et al., betulinic acid showed significant antiviral activity when tested in vitro on SARS-CoV-infected Vero E6 cells, inhibiting the replication of the virions at concentrations higher than 10 µM (EC_50_ value). Moreover, BA manifested no significant cytotoxic effect, only slightly interfering with the growth of the tested cells. Regarding its mechanism of action against SARS-CoVs, the authors noticed that, at the IC_50_ value of 10 µM, BA showed inhibitory effects on the main viral protease (3CLpro) [136], intervening in the viral replication [137]. Computer docking analysis revealed that BA can be nicely fitted into the substrate-binding pocket of SARS-CoV main protease [136,137], blocking its activity through competitive inhibition [136].

The BA inhibition mechanism of 3CLpro was associated with its molecular structure, as the hydroxyl group in the C3 position of BA is able to form a hydrogen bond with the oxygen atom of the carbonyl group of Thr24 located at the N-terminus of the protease [136]. This study demonstrated the antiviral activity of BA against SARS-CoVs in in vitro experiments, suggesting that it might be efficient against the newly emerged SARS-CoV-2, but further studies need to be conducted in this area. However, BA can be considered a potential adjuvant compound in treating COVID-19 infectious disease.

### 4.6. Anti-Inflammatory Compounds

The novel COVID-19 pathology is considered the consequence of a cytokine storm, an excessive and uncontrolled release of pro-inflammatory factors, leading to acute lung injury, acute respiratory distress syndrome, and death [47]. Therefore, anti-inflammation therapy might help prevent further aggravation of the disease. The pharmacological drug classes able to reduce inflammation include non-steroidal anti-inflammatory medication, glucocorticoids, chloroquine and hydroxychloroquine, immunosuppressants, and inflammatory cytokines antagonists (IL-6R monoclonal antibodies, TNF inhibitors, IL-1 antagonists, janus kinase inhibitor (JAK) inhibitors) [47].

Inflammation seems to be the most relevant process that increases virus-related organ damage and, subsequently, the severity of the pathology. Another relevant mechanism is considered the clathrin-mediated endocytosis. The main target is the numb-associated family of enzymes such as AAK1 and GAK because their inhibition reduces viral infection, interrupting the passage of the virus into the host cells [47]. As an example, baricitinib is a NAK inhibitor with high affinity for AAK1, acting as a strong anti-inflammatory drug in chronic inflammation in interferonopathies with favorable pharmacokinetic properties (low plasma protein binding, minimum interactions with CYP enzymes and, also drug transporters). It stands as an important example of a drug able to be associated with antiviral drugs in order to induce a higher efficacy in COVID-19 treatment. The most potent selective JAK inhibitors are baricitinib, fedratinib, and ruxolitinib; as a main activity, they are effective anti-inflammatory agents in arthritis and myelofibrosis. Their COVID-19 activity is related to the decrease in cytokine levels (including interferon-γ), often elevated in COVID-19 pathology. The most frequently reported side effects were related to upper respiratory tract infections. The only concern about the use of JAK inhibitors in COVID-19 therapy is the fact that they can inhibit a variety of inflammatory cytokines including INF-α, which plays a crucial role in fighting the virus [47].

Other compounds reported as potential anti-SARS-CoV-2 agents were those prescribed in oncology, sunitinib in combination with other antiarthrytic compounds—ruxolitinib and fedratinib. The mechanism of action includes the inhibition of enzymes involved in the clathrin-mediated endocytosis; tofacitinib is an example of drug that produces no inhibition of AAK1.

Important data from different in silico approaches to find inhibitors of SARS-CoV-2 were published in the last months. The “SARS-CoV E” protein sequence from NCBI (National Center for Biotechnology Information) database, Multalin, a web-based tool and PDB (Protein Data Bank), was used in a research based on molecular dynamic simulations (MDS) in its first phase, followed by the preparation of ligands, represented by 4153 phytochemicals from previous studies [138]; ligand’s topology parameters were generated during the second phase of MDS and inter-molecular interactions were performed at the end. The obtained results indicate that SARS-CoV-2 E is a pentameric protein comprised of 35 α-helices and 40 loops. Another similar study has begun with the RNA sequence of SARS-CoV-2 from NCBI database and uses the swiss model and PHYRE2 Protein Fold Recognition Server to discover the spike glycoprotein [139]; it was found that both the SARS-CoV-2 spike glycoprotein and ACE2-FC region of IgG1 present bonding and docking abilities.

Cava et al. [140] conducted an in silico investigation on the mechanism played by ACE2 in inflammatory lung disease to furnish some evidence for an inhibitor of SARS-CoV-2; the importance of this study lies in the fact that it is focused on the genes in the network that are already associated with known drugs such as nimesulide, didanosine, thiabendazole, fluticasone propionate, and Photofrin, and their role as a key treatment of COVID-19 is evaluated using a protein–protein interaction network containing the genes co-expressed with ACE2. Public data were extracted from The Cancer Genome Atlas Lung Adenocarcinoma and correlation analyses between ACE2 and 526 genes were performed; Pearson’s correlation with ACE2 expression level was also obtained. Their results indicate that nine genes (LRRK2, MCCC2, GSTA4, ACSL5, HSD1B4, EPHX1, ACACA, ROS1, and HGD) are positively correlated with ACE2 and Didanosine, a dideoxynucleoside analogue used in HIV treatment, has the highest antiviral activity [140]. Recent data on SARS-Cov-2 cases show that a relevant inflammatory cytokine storm is associated with disease severity [141]. Anakinra is a 17 kD recombinant, non-glycosylated human IL-1 receptor antagonist with a short half-life of about 3–4 h. The IL-1 receptor antagonist is a key treatment for hyperinflammatory conditions and was shown to be highly effective in the treatment of cytokine storm syndromes, such as macrophage activation syndrome and cytokine release syndrome [142]. Anakinra has a very safe profile and high dosages have been used even in patients with severe viral infections such as EBV, H1N1, and Ebola [143].

A recent cohort study evaluated the effect of anakinra on SARS-CoV-2-related hyperinflammatory state, in COVID-19 patients. The authors stated that anakinra significantly reduced both the need for invasive mechanical ventilation in the intensive care unit (ICU) and mortality among patients with severe COVID-19, without serious side-effects. The study also suggests that, so far, no other specific treatment has been shown to reduce the need for invasive mechanical ventilation and intensive care in patients admitted for COVID-19-associated pneumonia requiring oxygen therapy [144]. Up to this date, there are 10 ongoing clinical trials involving the use of anakinra as a COVID-19 potential therapy [145].

### 4.7. Immunotherapy

Immunotherapy is considered an effective method for the clinical treatment of infectious diseases. There are two main approaches in immunotherapy: passive immunization using antibodies isolated from the blood of the infected patients and the monoclonal antibodies (MAs) therapy and active immunization via vaccines [146]. The passive immunization by early administration of convalescent plasma or hyper-immune immunoglobulin from patients that contains significant antibody titers might be efficient in reducing the viral load and disease mortality, but there are some factors that need to be elucidated before treatment initiation, such as the availability of sufficient donors, clinical condition, viral kinetics, and host interactions of SARS-CoV-2 [12,35].

Considering the imperative need to find an effective treatment against SARS-CoV-2 infection (which is lacking at present), administration of immune “convalescent” sera containing neutralizing antibodies against SARS-CoV-2 was viewed as a viable approach, with rapid results by conferring immediate immunity to highly susceptible patients [147,148]. Convalescent serum/plasma exerts its highest efficacy if is administrated as prophylaxis or early after the onset of the clinical symptoms [147,148]. The data regarding convalescent plasma administered as treatment in COVID-19 patients are rather scarce, and only several reports from China assert the efficacy of this kind of treatment (small size studies) characterized by an improvement of clinical outcome, decreased viral loads, and clinical stabilization [147].

The use of convalescent plasma for COVID-19 therapy was also considered an optimal alternative by the FDA, which, on 24 of March 2020, published the guidance for Investigational COVID-19 Convalescent Plasma that highlights the access pathways to this kind of treatment: (1) for compassionate use in severely or critically ill patients; (2) for clinical trials settings; and (3) to institutions that participate in a master treatment protocol—a government led-initiative [147,149]. To gather more information about convalescent plasma efficacy/safety profile, multiple clinical trials (even phase 2) were set in motion and assessed the following directions: (i) treatment for mild forms of SARS-CoV-2 infection; (ii) treatment for moderately ill patients; (iii) as rescue intervention conducted in patients that are mechanically ventilated; (iv) safety and pharmacokinetics in high-risk pediatric patients; and (v) as post-exposure prophylaxis in patients that were in contact with COVID-19 positive patients, but do not present clinical symptomatology [147].

The risks associated with convalescent plasma administration can be classified in two categories: known, which include immunological reactions (serum sickness), transfer of other infectious agents, and allergic reactions to serum constituents; and theoretical, which comprise development of antibody-dependent enhancement of infection phenomenon, prevention of the infection via a pathway that reduces the immune response, and these patients become susceptible to subsequent reinfection [147,148]. Further clinical studies are required to confirm the convalescent plasma effectiveness and safety profile.

Regarding monoclonal antibodies (MAs), they form a versatile class of pharmaceuticals able to provide an efficient and highly specific treatment against diseases, including viral infections. The monoclonal antibodies could effectively block the virus entry within the host cell, binding either the spike (S) protein or the ACE2 receptor. Even though there are several reported MAs targeting the RBD region of S protein, a receptor-binding domain located in the S1 subunit that mediates the virus attachment to the host cells, the large-scale production of monoclonal antibodies is labor-intensive, expensive, and time-consuming, which outweighs their clinical application especially during emerging situations like the novel SARS-CoV-2 outbreak [12,35].

An example of monoclonal antibody included in the interim guidelines for COVID-19 treatment in critically ill patients is tocilizumab. This agent is a humanized monoclonal antibody that acts as an inhibitor of interleukin-6 and is currently used in cytokine release syndrome. The mechanism of action is related to its capacity to inhibit IL-6, a pro-inflammatory cytokine, leading to a decrease in intensity of the inflammatory status developed by the critically ill COVID-19 patients. The existent clinical data concerning tocilizumab efficacy report encouraging results, such as a significant decrease of inflammatory markers, radiological improvement, and decreased ventilatory support. In terms of safety profile, even though several adverse reactions were described, the directly-linked effects to tocilizumab use were an increase of hepatic enzymes values and development of opportunistic infections owing to its immunomodulating activity; the other effects were correlated with the drugs coadministered (anemia–ribavirin, QT interval prolongation–hydroxychloroquine) [150].

According to the drug–drug interactions study conducted by Liverpool University for anti-SARS-CoV-2 drugs, tocilizumab should not be administered with the following agents: metamizole (high risk of hematological toxicity), immunosuppressants (adalimumab and basiliximab), and lipid lowering agents (evolocumab) [80]. Coadministration with other investigated agents for COVID-19 therapy such as chloroquine, hydroxychloroquine (potential additive toxicity), and ribavirin (risk of hematological toxicity) requires close monitoring and possible dose adjustment. No interactions were reported between tocilizumab and lopinavir/ritonavir or remdesivir [80].

Type I interferons (IFN), especially IFN-beta, have been proposed as potential cornerstone therapies to address severe COVID-19 and are currently assessed in REMAP-CAP and the WHO’s Solidarity Trial [151]. An important perspective consists of the fact that COVID-19 pathology induces an excessive IFN-I mediated antiviral response, leading to tissue damage. Therefore, IFN-I treatment should be limited to the early phases of the infection if this hypothesis is confirmed, as shown by previous studies [152] and by early clinical data suggesting a link between inflammatory biomarkers and increased mortality [153].

A recent non-controlled prospective trial (IRCT20151227025726N12) evaluated the subcutaneous use of INF beta-1a in combination therapy with hydroxychloroquine and lopinavir/ritonavir. The early results showed a positive response in terms of symptomatology (mainly fever) resolution, virologic clearance, and hospitalization period [154]. However, some authors debate the need to address the clear differences concerning s.c versus i.v. administration, as these routes significantly influence the bioavailability of the drug and, consequently, the therapeutic response [151].

On the basis of previous results, an open-label, randomized, phase 2 trial (NCT04276688) evaluated the clinical impact of the triple combination, IFN beta-1b, lopinavir–ritonavir, and ribavirin [155]. The authors showed that treatment with the triple combination therapy effectively suppressed viral load in all clinical admitted patients, in most cases, within 8 days from treatment commencement, a significantly shorter period compared with the time taken in the control group. The results also revealed that the triple combination also alleviated symptoms completely in a significantly shorter time (4 days) compared with the control and suppressed IL-6 levels [155].

### 4.8. Anticoagulant Therapy

On the basis of the latest preliminary reports (retrospective analyses with a reduced number of patients), COVID-19 infection was adjoined with an increased susceptibility of patients to develop thrombotic events characterized by hemostatic disturbances (most common mild thrombocytopenia and augmented level of D-dimer) and even disseminated intravascular coagulation (DIC). It is still unknown if the hemostatic disorders are a cause of SARS-CoV-2 infection or a repercussion of the cytokine storm that triggers the beginning of inflammatory response syndrome (SIRS). Another hypothesis is a possible link between the hemostatic changes and liver dysfunction [41].

An explanation for the thrombotic events in COVID-19 could lie in the impairment of the vascular endothelial cells (which present a high expression of ACE2 receptors on their surface) by the viral infection [36], which triggers a hypercoagulable state by an increased thrombin production and suppressed fibrinolysis; the final result is the coagulopathy dysfunction described as one of the main causes for the death of severe ill COVID-19 patients [41,156]. 

The World Health Organization interim guidance statement indicates daily administration of low-molecular weight heparins (LMWHs) or twice daily subcutaneous unfractionated heparin (UFH) for the prophylaxis of thrombotic events in COVID-19 patients [41].

The use of LMWHs as anticoagulant therapy in COVID-19 infection is controversial at this time; the Chinese recommend the use of heparin (which also exerts an anti-inflammatory effect) as early anticoagulant treatment in severely ill patients in order to prevent disseminate intravascular coagulation and venous thrombosis, as signs of these disturbances were observed in pulmonary small vessels of critically ill COVID-19 patients (occlusion and microthrombosis), whereas the Japanese guidelines are against the use of heparin or heparin analogs in septic associated coagulopathy [156].

The International Society on Thrombosis and Haemostasis (ISTH) proposed a guide for anticoagulant therapy in sepsis-induced coagulation, so that only the patients that meet the criteria should receive the treatment. The onset of the therapy is a key element because coagulation has the role of isolating the virus and decreasing its invasion, and the administration of anticoagulants to patients with no risk to develop these events might instead determine a spread of the virus into the body and aggravation of the patient clinical status [156,157].

The reluctance in recommending LMWHs as a treatment for thromboembolic disease in severely ill COVID-19s patient is based on the pharmacokinetic profile of these drugs; that is, a long half-life, which makes it difficult to monitor their dosage, as well as an elevated risk to produce heparin-induced thrombocytopenia (a severe adverse event). It is thus recommended that LMWhs be administered in mild and moderate coagulation impairment, whereas in severe conditions, the unfractionated heparin should be the first choice for intravenous administration (short half-life, can be easily monitored, and can be inactivated by protamine). An alternative in the case of adverse effects (heparin-induced thrombocytopenia) after heparin treatment is represented by argatroban, a direct thrombin inhibitor, or bivaluridin, a direct and specific inhibitor of thrombin activity [158].

According to the COVID-19 drug interactions study developed by Liverpool University, both heparin and argatroban are not susceptible to determine interactions with the investigational anti-COVID-19 agents (RDV, LPV/r, CQ, and HCQ) [80].

## 5. Closing Remarks and Future Perspectives

The outbreak determined by the newly emerged coronavirus, SARS-CoV-2, has initiated a roller effect both at medical and industrial levels, and its consequences are already seen in different areas. It has been more than five months since the first mention of this virus new potential, and if, at the beginning, there were more missing pieces, the latest data managed to almost complete the puzzle, as follows: (i) SARS-CoV-2 has a zoonotic origin (bats are considered the primary source); (ii) the genomic structure of the virus was elucidated, leading to the development of diagnostic tools and of potential novel innovative antivirals; (iii) human infection requires the binding of S spike glycoprotein to the human ACE2 receptor expressed by epithelial respiratory cells, vascular endothelial cells, cardiomyocytes, gastrointestinal cells, and hepatocytes, among others; (iv) human-to-human transmission occurs via respiratory droplets, direct contact, fecal–oral route, environmental transmission, and bodily fluids (key data for the infection spread repression); and (v) the clinical impact of SARS-CoV-2 infection (characteristic symptomatology, onset, clinical stages, complications) is mostly described, but the data in this area are constantly being updated.

The urge to find a therapy for SARS-CoV-2 infection determined the occurrence of multiple therapeutic alternatives, such as repurposed drugs, broad-spectrum antivirals (remdesivir, lopinavir/ritonavir, oseltamivir, and so on), antimalarial drugs (chloroquine and hydroxychloroquine), anti-inflammatory compounds (baricitinib, fedratinib, ruxolitinib, sunitinib), anticoagulants (low molecular weight heparin, unfractionated heparin), convalescent plasma, and novel potential antivirals (vaccines, anti-SARS-CoV-2 antibodies, natural compounds, and so on).

At present, remdesivir is considered the most promising therapy for SARS-CoV-2 infection being already included in the interim guidelines for COVID-19 treatment, based on the following considerations: proved to be an effective drug in severely and critically ill forms of COVID-19; shows a high human tolerance; the only side effect directly correlated to RDV is the hepatic injury (elevation of hepatic enzymes values); has a very low potential to induce drug–drug interactions; and the duration of treatment can be reduced at 5 days according to the latest clinical results. Although the mechanism of action of RDV is well defined, its toxicological profile needs further investigations in order to be fully established, so the administration of this compound should be performed under surveillance.

Chloroquine and hydroxychloroquine are recommended for compassionate use in mild-to-moderate (as single treatment option) and in severely and critically ill forms of COVID-19 (as comedication with remdesivir or lopinavir/ritonavir). These drugs present efficient oral absorption and distribution patterns, several drug–drug interactions were described (see detailed list at https://www.covid19-druginteractions.org/), and a thorough risk/benefit ratio analyze should be conducted before their administration mainly in combination with lopinavir/ritonavir (might increase the risk for prolongation of QT interval). Still, their clinical effectiveness as anti-COVID-19 agents is debatable at this point.

The use of lopinavir/ritonavir against SARS-CoV-2 infection determined controversial results; that is, according to some clinical studies, this combination had no significant antiviral effect, whereas in terms of adverse events and drug–drug interactions, the significance was reached (both lopinavir and ritonavir are CYP3A inhibitors and are subjected to multiple drug–drug interactions and adverse effects implicitly). Therefore, lopinavir/ritonavir is recommended as a second choice in the interim guidelines for remdesivir and its administration should be performed only under strict surveillance. Administration of anti-inflammatory agents (baricitinib, fedratinib, sunitinib, ruloxitinib, and so on) should be recommended as therapy for SARS-CoV-2 infection only when the laboratory results indicate a potential “cytokine storm” occurrence. The initiation of anticoagulant treatment should be performed based on laboratory results that indicate a potential risk for thrombotic events and coagulopathy disfunction; the administration of LMWHs is recommended in mild and moderate coagulation impairment, whereas in severe conditions, unfractionated heparin should be the first choice.

Other compounds described in our manuscript such as oseltamivir, ribavirin, arbidol, and natural compounds (betulinic acid) proved to have potent in vitro antiviral activity against SARS-CoV-2; still, further studies (preclinical and clinical) are required to confirm their effectiveness as anti-COVID-19 therapeutic agents. Convalescent plasma collected from COVID-19 patients represents a promising therapeutic alternative with immediate results; however, some limitations were noticed in terms of its obtaining and approval for use (the ongoing clinical trials results will confirm its effectiveness).

In defiance of the great efforts recorded, no drug was approved as a specific anti-SARS-CoV-2 treatment up to present. The considerable number of ongoing clinical trials (over 1300) that evaluate multiple potential antivirals represent the future perspectives concerning the elucidation of COVID-19 pathology and finding an appropriate treatment. The information regarding the progress recorded in the field of vaccine development is also optimistic and represents an option of selective and potent intervention.

## Figures and Tables

**Table 1:** Interim coronavirus disease (COVID-19) therapeutic guidelines in different countries dependent on clinical stage.

Clinical Status	Belgium	Italy (Lombardia Protocol)	France	Netherlands	Switzerland	Romania
Mild-to-moderate—no risk group	Symptomatic care (paracetamol) No antiviral treatment	No antiviral treatment	No antiviral treatment	No antiviral treatment	No antiviral treatment	Ambulatory symptomatic care—paracetamol—500 mg/3x/day Hospital treatment—HCQ—400 mg/2x/day—only on day 1 and 200 mg/2x/day—5–6 days Second choice—LPV/r—400/100 mg/2x/day—5–7 days
Mild-to-moderate—risk group	HCQ 400 mg at suspicion/diagnosis; 400 mg—12 h later and 200 mg—until day 5—in the absence of HCQ consider CQ base (600 mg/diagnosis; 300 mg—12 h later and 300 mg up to 5 days) or CQ phosphate (1000 mg/diagnosis; 500 mg—12 h later and 300 mg up to 5 days)	LPV/r (400/100 mg/BD) + CQ (500 mg/BD) or HCQ (200 mg/BD)—5–7 days	LPV/r (400/100 mg/BD) (under consideration) Treatment period—depending on the viral excretion	CQ—5 days (under consideration day 1: 600–300 mg; days 2–5: 300 mg)	Not mentioned	HCQ (400 mg/2x/day—only on day 1 and 200 mg/2x/day—4 days) + LPV/r (400/100 mg/2x/day)—10–14 days
Severe disease	HCQ—400 mg at suspicion/diagnosis; 400 mg—12 h later and 200 mg—until day 5—in the absence of HCQ consider CQ base (600 mg/diagnosis; 300 mg—12 h later and 300 mg up to 5 days) or CQ phosphate (1000 mg/diagnosis; 500 mg—12 h later and 300 mg up to 5 days) Second option: LPV/r 400/100 mg (=2 tablets of 200/50 mg) BD for 14 days	RDV (200 mg/day—day 1 followed by 100 mg/day days 2–10) + CQ (500 mg/BD) or HCQ (200 mg/BD)—5–20 days (in the absence of RDV, it can be maintained LPV/r+ CQ)	RDV (200 mg/day—day 1 followed by 100 mg/day days 2–10) Treatment period—depending on the viral excretion No second option	CQ—5 days (day 1: 600–300 mg; days 2–5: 300 mg) LPV/r (400/100 mg/BD) as second choice—10–14 days	LPV/r (400/100 mg/BD) (atazanavir/ritonavir as second option)	HCQ (400 mg/2x/days—only on day 1 and 200 mg/2x/day—4–20 days) + RDV (200 mg/day—only on day 1, followed by 100 mg/day for other 9 days) Second choice for RDV is LPV/r, but only until RDV is obtained. ± Tocilizumab (only to patient that present “cytokine storm” and organ dysfunctions)—8 mg/kg body weight (maximum 800 mg)—lent perfusion—1–3 doses at 8 h interval
Critical disease	RDV (compassionate use) 200 mg loading dose (IV, within 30 min) 100 mg OD for 2 to 10 days In the absence of RDV—(HCQ, crushed in nasogastric tube, at the same dosage)	RDV (200 mg/day—day 1 followed by 100 mg/day days 2–10) + CQ (500 mg/BD) or HCQ (200 mg/BD)—5–20 days (in the absence of RDV, it can be maintained LPV/r+ CQ)	RDV (200 mg/day—day 1 followed by 100 mg/day days 2–10) Treatment period—depending on the viral excretion LPV/r—as second option (case by case)	RDV (for 10 days—200 mg/day—day 1 followed by 100 mg/day days 2–10) + CQ (for 5 days—day 1: 600–300 mg; days 2–5: 300 mg)	RDV—10 days (200 mg/day—day 1 followed by 100 mg/day days 2–10) LPV/r (400/100 mg/BD) (+HCQ if <65 years/no comorbidity) as second choice (if RDV is unavailable). Tocilizumab (in the case of MOF and inotropic support)	

HCQ—hydroxychloroquine; CQ—chloroquine; LPV/r—lopinavir/ritonavir; RDV—remdesivir; MOF—multiorgan failure; BD—twice a day; IV–intravenous; OD–once a day.

**Table 2:** Brief description of COVID-19 therapeutic options recommended by World Health Organization (WHO) guidelines [117,118,119,120].

Drug Name	Pharmacological Class	Clinical Phase	EC_50_ (half maximal effective concentration)	Dose	Mechanism of Action	Adverse Effects
**Remdesivir—(RDV)**	nucleoside analogue	Severe	0.77 µM	200 mg—day 1; 100 mg/day—9 days	inhibitor of the CoVs RNA-dependent RNA polymerase (RdRp)	incompletely characterized toxicological profile: phlebitis, constipation, headache, ecchymosis, nausea, pain in the extremities elevation of hepatic enzymes values
**Chloroquine (CQ)**	4-aminoquinoline	Mild-to-moderate and severe—depending on the guideline applied	23.90 µM (24 h) 5.47 µM (48 h)	CQ base (600 mg/diagnosis; 300 mg—12 h later and 300 mg up to 5 days) or CQ phosphate (1000 mg/diagnosis; 500 mg—12 h later and 300 mg up to 5 days)	weak base able to elevate the pH of acidic intracellular organelles, such as endosomes and lysosomes	retinopathy hypotension ECG changes irreversible cardiomyopathy—long-term users direct myocardial toxicity exacerbate the existent myocardial dysfunction QT prolongation risk of Torsade de Pointes (TdP) even at therapeutic doses interaction with antiarrhythmics * in the case of HCQ, the adverse effects have a lower intensity, but are not absent
**Hydroxychloroquine (HCQ)**	4-aminoquinoline	Mild-to-moderate and severe—depending on the guideline applied	6.14 µM (24 h) 0.72 µM (48 h)	HCQ—400 mg at suspicion/diagnosis; 400 mg—12 h later and 200 mg—until day 5	weak bases able to elevate the pH of acidic intracellular organelles, such as endosomes and lysosomes	
**Liponavir/ritonavir (LPv/r)**	Protease inhibitor	Mild-to-moderate	-	400 mg/100 mg/day—14 days	peptidomimetic inhibitor of HIV protease enzyme	hypercholesterolemia and increased serum triglycerides increased gamma-glutamyl transferase increased serum ALT upper respiratory tract infection diarrhea nausea headache skin rush neutropenia anxiety QT prolongation
